# Salicylic acid is an indispensable component of the *Ny-1* resistance-gene-mediated response against *Potato virus Y* infection in potato

**DOI:** 10.1093/jxb/ert447

**Published:** 2014-01-13

**Authors:** Š. Baebler, K. Witek, M. Petek, K. Stare, M. Tušek-Žnidarič, M. Pompe-Novak, J. Renaut, K. Szajko, D. Strzelczyk-Żyta, W. Marczewski, K. Morgiewicz, K. Gruden, J. Hennig

**Affiliations:** ^1^National Institute of Biology, Večna pot 111, 1000 Ljubljana, Slovenia; ^2^Institute of Biochemistry and Biophysics, Polish Academy of Sciences, Pawińskiego 5a, 02-106 Warsaw, Poland; ^3^Centre de Recherche Public—Gabriel Lippmann, 41, Rue du Brill, L-4422 Belvaux, Luxembourg; ^4^Plant Breeding and Acclimatisation Institute—National Research Institute, Platanowa 19, 05-831 Młochów, Poland

**Keywords:** Plant–pathogen interactions, *Potato virus Y*, *Potyviridae*, salicylic acid, *Solanum tuberosum*, whole transcriptome analysis.

## Abstract

The purpose of the study was to investigate the role of salicylic acid (SA) signalling in *Ny-1*-mediated hypersensitive resistance (HR) of potato (*Solanum tuberosum* L.) to *Potato virus Y* (PVY). The responses of the *Ny-1* allele in the Rywal potato cultivar and transgenic NahG-Rywal potato plants that do not accumulate SA were characterized at the cytological, biochemical, transcriptome, and proteome levels. Analysis of noninoculated and inoculated leaves revealed that HR lesions started to develop from 3 d post inoculation and completely restricted the virus spread. At the cytological level, features of programmed cell death in combination with reactive oxygen species burst were observed. In response to PVY infection, SA was synthesized *de novo*. The lack of SA accumulation in the NahG plants led to the disease phenotype due to unrestricted viral spreading. Grafting experiments show that SA has a critical role in the inhibition of PVY spreading in parenchymal tissue, but not in vascular veins. The whole transcriptome analysis confirmed the central role of SA in orchestrating *Ny-1*-mediated responses and showed that the absence of SA leads to significant changes at the transcriptome level, including a delay in activation of expression of genes known to participate in defence responses. Moreover, perturbations in the expression of hormonal signalling genes were detected, shown as a switch from SA to jasmonic acid/ethylene signalling. Viral multiplication in the NahG plants was accompanied by downregulation of photosynthesis genes and activation of multiple energy-producing pathways.

## Introduction

Potato (*Solanum tuberosum* L.) is the fourth-most-important food crop in the world, with a yield of 324 million tonnes in 2010, and a continuous increase in the world production of tubers (http://faostat.fao.org/). *Potato virus Y* (PVY) is a member of the Potyviridae family, and it is currently considered as the most economically harmful virus for cultivated potatoes. PVY has a wide host range, mainly within the Solanaceae family, and it is distributed worldwide. Isolates of PVY species are highly variable at the biological, serological, and molecular levels (Scholthof *et al.*, 2011). Strain PVY^N-Wi^, which was originally described in the Polish cv. Wilga (Chrzanowska, 1991), belongs to the N strain group, although it is serologically related to PVY° (Singh *et al.*, 2008). In the current context of a highly competitive international potato market, the weaknesses in both the knowledge of PVY–host interactions and the diagnostic tools have led to a situation in which necrotic PVY isolates are still potentially responsible for huge agronomic and economic losses (Scholthof *et al.*, 2011).

The ability of viruses to cause disease is determined by molecular interactions between the host plant and virus factors. These interactions directly affect virus replication and movement, symptom development, and host defence responses, leading to either compatible (sensitive) or incompatible (resistant) interactions (Whitham *et al.*, 2006). In potato, there are two main types of resistance to PVY: extreme resistance, conferred by the *Ry* genes, and hypersensitive resistance (HR), conferred by the *Ny* genes (reviewed in Kogovšek and Ravnikar, 2013). The potato cv. Rywal carries the *Ny-1* gene, and it develops HR that is manifested as necrotic lesions on inoculated leaves 3 d post inoculation (dpi) with various PVY strains (PVY°, PVY^N^, PVY^N–Wi^, PVY^NTN^). Development of HR restricts virus multiplication and spreading. However, the response is temperature dependent, as growth at a higher temperature (28 °C) prevents HR and allows the virus to spread systemically (Szajko *et al.*, 2008).

Salicylic acid (SA) is a key component in the orchestration of the events during and following HR (Lewsey *et al.*, 2009). Deficiency in SA signalling leads to an impairment of the defence responses and susceptibility to pathogen attack (Takahashi *et al.*, 2004; Sanchéz *et al.*, 2010; Jovel *et al.*, 2011). In the case of compatible interactions, SA can still improve the immunity by delaying the onset of viral replication and disease development, through mediation of the expression of a cohort of defence-related genes that induce a defence-like response (Huang *et al.*, 2005; Baebler *et al.*, 2011). Previous studies have suggested significant differences in the possible mechanism of SA action in potato defences in comparison to the model pathosystems of *Arabidopsis thaliana* and tobacco. In potato, basal levels of salicylates are 10–100-fold higher than in *Arabidopsis* or tobacco (Navarre and Mayo, 2004), and the increase in SA levels after pathogen treatment is relatively moderate (Krečič-Stres *et al.*, 2005; Halim *et al.*, 2007). Therefore, it has been suggested that potato has poor SA sensitivity and that, rather than biosynthesis, enhancement of its effectiveness is responsible for activating the SA-dependent signal transduction pathway after infection (Yu *et al.*, 1997). It has been proposed that the inactive salicylate glycosylates (SAGs) are the storage form of salicylates that, when necessary, can be converted into the free and active SA (Hennig *et al.*, 1993). Plants that are unable to accumulate SA due to the expression of salicylate hydroxylase (*NahG*), which catalyses the conversion of SA to catechol, have been widely used to study the role of SA in potato–pathogen interaction (Navarre and Mayo, 2004; Halim *et al.*, 2007; Yu *et al.*, 1997; Sanchéz *et al.*, 2010; Baebler *et al.*, 2011).

The understanding of the mechanisms that underlie resistance responses to viruses is still incomplete. The network of factors that can interact to establish resistance has to be of great complexity to provide a plant with effective weaponry to fight the variety of pathogens. Genomic tools have allowed the assessment of gene expression at a genome-wide scale, which has provided unprecedented views of host–virus interactions (Rodrigo *et al.*, 2012). Compared to numerous whole transcriptome studies on compatible plant–virus interactions, which were reviewed and subjected to meta-analysis by Postnikova and Nemchinov (2012) and Rodrigo and coworkers (2012), studies of incompatible interactions are scarce (Ishihara *et al.*, 2004; Marathe *et al.*, 2004; Baebler *et al.*, 2009; Vuorinen *et al.*, 2010; Pacheco *et al.*, 2012). As far as is known, there has been only one whole-transcriptome approach to investigate the involvement of SA in the response of *A. thaliana* to a set of compatible viruses (Huang *et al.*, 2005), while the role of SA in potato–virus interactions has not been investigated yet at the whole-transcriptome level.

The aim of the present study was to investigate the role of SA signalling in *Ny-1*-mediated HR. The responses to PVY^N–Wi^ infection were studied in a *Ny-1* background in nontransgenic plants, plants impaired in SA accumulation, and plants with compromised resistance due to its temperature-dependent nature. Cytological and biochemical characterization combined with transcriptome and proteome analyses have revealed that SA is a crucial factor for inhibition of the spread of PVY in parenchymal tissue, while a lack of SA results in delayed early transcriptional events, which can lead to inefficient defence responses and disease development.

## Materials and methods

### Plant material

Potato (*S. tuberosum* ssp. *tuberosum*; 2n=4x=48) cv. Rywal plants were obtained from the Institute of Plant Breeding and Acclimatisation—National Research Institute, Młochów (Poland). Plants expressing the *NahG* transgene (NahG-Rywal) were generated using the binary plasmid pCIB containing salicylate hydroxylase (*NahG*), which was generously provided by Syngenta Biotechnology. The plasmid was introduced into *Agrobacterium tumefaciens* LBA4404 and used for potato leaf disc transformation (Chen *et al.*, 1994). Transgenic potato plants were regenerated as described by Mac *et al.* (2004). Plants of both genotypes were grown for 4 weeks in soil under controlled environmental conditions (16/8 light/dark cycle, 20 °C) as described previously (Szajko *et al.*, 2008). PVY strain N-Wilga (PVY^N–Wi^; accession no. EF558545) was derived from potato cv. Wilga and was multiplied and maintained in tobacco plants, *Nicotiana tabacum* cv. Samsun.

PVY inoculation was performed on 4-week-old potato plants. Three bottom leaves were dusted with carborundum powder and rubbed with cheesecloth dipped in a sap prepared from the leaves of the PVY-infected tobacco plants. After 10min, the leaves were washed liberally with tap water. In mock inoculations, water was used instead of the sap.

Viral amplification was monitored by semiquantitative reverse-transcription PCR as described before (Szajko *et al.*, 2008), but by running only 22 cycles. Separate PCR reactions of potato elongation factor 1-α (*EF-1*) expression were performed as loading control (Varet *et al.*, 2002).

### Quantification of free salicylic acid and salicylic acid glycosylates

Free SA was extracted and quantified from noninoculated and inoculated leaves at several times after mock or viral inoculation, as described previously (Malamy *et al.*, 1990) and with modifications introduced by Krzymowska *et al.* (2007). HPLC analysis was performed and SAGs were quantified as described previously (Malamy *et al.*, 1992). Total salicylates were calculated as the sum of the free SA and SAGs.

### Callose and hydrogen peroxide staining

Visualization of callose deposition in leaf discs was performed according to Peterhansel *et al.* (1997) and Vogel and Somerville (2000). Hydrogen peroxide (H_2_O_2_) was visualized using 1mg ml^–1^ 3,3′-diaminobenzidine-HCl, pH 3.8 (DAB), as described previously (Thordal-Christensen *et al.*, 1997; Talarczyk *et al.*, 2002). Microscopic observations and documentation were performed using Microphot-SA fluorescence microscope (Nikon Corporation) with a OCRA–ER digital camera (Hamamatsu Photonics).

### Light and transmission electron microscopy

For morphological and ultrastructural observations, fragments of the leaves were dissected from mock-inoculated and PVY-inoculated potato plants, at 0, 1, 3, and 6 dpi. The leaf pieces were fixed in freshly made Karnovsky fixative for 2h under reduced pressure (0.4 atm). They were then post-fixed in 2% (w/v) osmium tetroxide in 0.05M sodium cacodylate buffer for 2h and embedded in EPON epoxy resin (Fluka). Semithin sections were stained with crystal violet (1% w/v) and inspected under an Olympus AX70 microscope, with the images captured with an Olympus DP50 digital camera. For ultrastructure observations, ultra-thin sections were stained with uranyl acetate and lead citrate, and examined with a JEM 1200 EX transmission electron microscope (JEOL).

The presence of viral particles in inoculated leaves of cv. Rywal was examined 6 dpi by the negative-staining method under transmission electron microscopy (Supplementary Fig. S1, available at *JXB* online).

### Microarray analysis

Total RNA from the inoculated leaves was extracted, treated, purified, and quality controlled as described previously (Baebler *et al.*, 2009). The custom 60-mer oligo microarrays (4×44; design ID 015425; Agilent) were designed by the Potato Oligo Chip Initiative consortium (Kloosterman *et al.*, 2008). For microarray hybridization, 1 μg RNA was spiked with 500ng One-Color Spike-Mix (Agilent), amplified and labelled with Cy-5 using MessageAmp II aRNA amplification kit (Ambion), and hybridized using Gene Expression Hybridization kits (Agilent), according to manufacturer protocols. The dried microarrays were scanned with an LS200 Array Scanner (Tecan) and the images were analysed and data extracted with ArrayPro (Media Cybernetics), according to Baebler *et al.* (2009).

The data were analysed in the R environment (R Core Team, 2012) with the Bioconductor limma package (Smyth, 2005). The background signal on all of the microarrays was uniform and low and was hence ignored in further calculations. The raw data was quantile normalized. Features below background intensity (log_2_A <5) in more than 95% of samples were excluded from further analysis. The correlation coefficients between biological replicates ranged from 0.97 to 1.00, indicating their high similarities. Differentially expressed genes (Benjamini and Hochberg corrected *P*-value ≤0.05, fold change ≥2) between virus and mock-inoculated plants at each time point and for each genotype were identified using the empirical Bayes method (Smyth, 2005). Functional analysis of differentially expressed genes was performed using MapMan ontology (Usadel *et al.*, 2009), adapted for potato (Rotter *et al.*, 2007). Gene set enrichment analysis (GSEA; Subramanian *et al.*, 2005) was performed to search for groups of genes involved in the same processes that were significantly (false discovery rate corrected *Q*-value < 0.05) altered by virus inoculation, using MapMan ontology as the source of the gene sets.

The data discussed in the present study have been deposited in NCBI Gene Expression Omnibus (Edgar *et al.*, 2002) and are accessible through GEO Series accession number GSE46180.

To validate the microarray results, the expression of the genes involved in carbohydrate metabolism, hormonal signalling, and defence responses was analysed by quantitative PCR in the same RNA samples as for microarray analysis (Supplementary Table S1).

### Two-dimensional differential gel electrophoresis

To complement the data at the transcriptome level, proteome analyses were performed on cv. Rywal, PVY, and mock-inoculated leaves, collected 1 and 3 dpi, and taken from the same plants (four biological replicates), as for the transcriptome analysis. Protein extraction and separation were carried out as described previously (Renaut *et al.*, 2008) using 300mg fresh leaf material. A statistical analysis of spot abundances was performed using the same software: t-test with *P*<0.05 and abundance variation (infected/mock) of at least 1.3-fold; two-way ANOVA was performed to find proteins differentially expressed in time after inoculation (*P*<0.05, PVY infection as one factor, time as the second factor). Differentially expressed spots were excised from gels, digested with trypsin, and spotted on MALDI targets, as described previously (Renaut *et al.*, 2008). Proteins were identified by searching against the NCBI nr (Viridiplantae) and potato EST databases using a MASCOT server (Matrix Science, London; www.matrixscience.com). Searches were carried out using a mass window of 150 ppm for the precursor and 0.75Da for the fragments. The search parameters allowed for carboxyamidomethylation of cysteine, oxidation of methionine, tryptophan to kynurenine, and double oxidation of tryptophan (N-formylkynurenine). The search results were evaluated based on the peptide scores. Homology identification was retained with a probability set at 95%. Proteins were identified with at least two unique peptides with a significant score.

## Results

### Morphological characterization of HR response to PVY

**Fig. 1.**
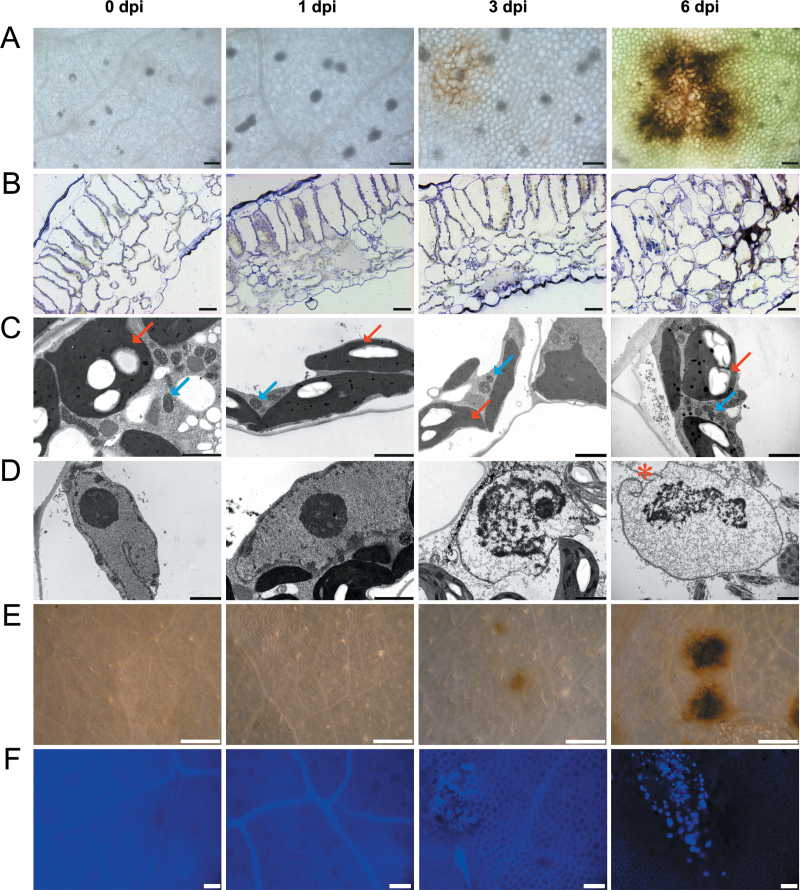
Analysis of the hypersensitive response to PVY infection at the macroscopic, light and electron microscopy levels in tissue samples from PVY-inoculated cv. Rywal leaves at 0, 1, 3, and 6 dpi. (A) Chlorophyll-removed tissue under the dissecting microscope. (B) Cross-sections of leaf tissue fixed for electron microscopy. (C, D) Transmission electron micrographs. (E) DAB-stained tissue under the dissecting microscope. (F) Callose-stained tissue observed under the fluorescence microscope with a DAPI filter. Arrows in C, chloroplasts (red), mitochondria (blue); asterisk in D, nuclear envelope invagination. Bars: 100 μm (A, F), 20 μm (B), 2 μm (C, D), 1mm (E).

To understand HR response observed in cv. Rywal, lesion development was examined at the cytological level. Lesions started to appear on the abaxial side of the leaf in mesophyll cells at 3 dpi and became fully developed at 6 dpi. Deposition of brown-coloured material, probably containing phenolic compounds (e.g. lignins) was observed at the lesion edge at 6 dpi (Fig. 1A). Disorganization of tissue architecture at the site of the lesion was observed under light microscopy (Fig. 1B). Analysis of the cells in this area by electron microscopy revealed no significant changes in the sizes and shapes of the chloroplasts or mitochondria (Fig. 1C, arrows). In contrast, the nuclei and nucleoli underwent gradual degradation, which was already visible at 3 dpi and was even more pronounced at 6 dpi (Fig. 1D). At 3 dpi, the nucleoli were significantly smaller in comparison to the 0 and 1 dpi samples, and at 6 dpi the nucleoli appeared to be partially degraded. At 3 and 6 dpi, the chromatin was partially condensed. At 6 dpi, nuclear envelope invaginations were visible (Fig. 1D, red asterisk).

DAB staining revealed high levels of H_2_O_2_ in the areas covering and surrounding the lesions (Fig. 1E, 3 and 6 dpi), which suggests the involvement of reactive oxygen species (ROS) in this HR process. Also, callose deposits (detected by aniline blue staining) were present in the lesion areas at 3 dpi and were even more abundant at 6 dpi (Fig. 1F).

Negative-staining electron microscopy detected the presence of PVY viral particles in the extracts of the inoculated leaves at 6 dpi, as isolated from the lesion areas (Supplementary Fig. S1), but not in extracts from the surrounding tissues. This confirms that the HR completely restricted the viral spread.

### Dynamics of SA accumulation in HR response to PVY

**Fig. 2.**
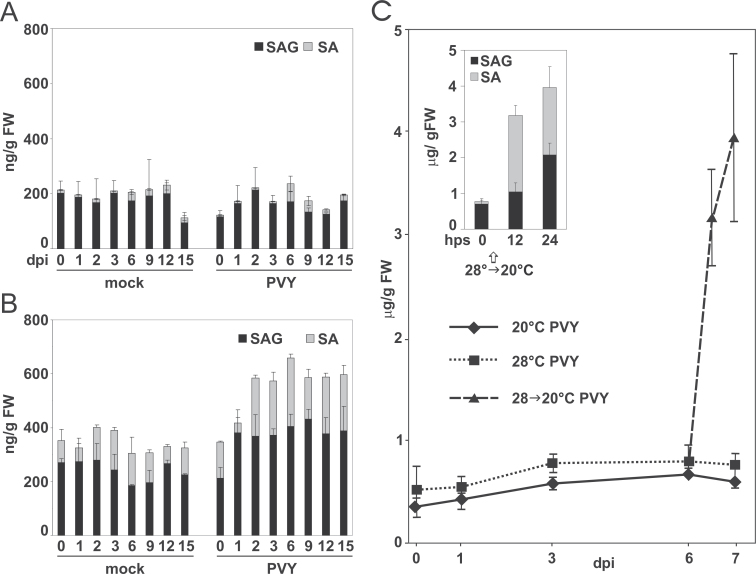
Changes in SA levels after PVY inoculation. (A, B) Changes in SA and SAG levels after mock or PVY inoculation in upper noninoculated (A) and inoculated (B) leaves of potato cv. Rywal from 0 to 15 d post infection (dpi). (C) Total salicylates in PVY-inoculated leaves, at stable temperatures (20 and 28 °C) and with temperature shift from 28 °C to 20 °C at 6 dpi. Inset: free SA and SAG at 0, 12, and 24h post temperature shift (hps). Data are mean ± standard deviation (*n*=5). SA, salicylic acid; SAG, salicylate glycosylates. Statistical significance of data is presented in Supplementary Table S1.

To study the role of SA in the response of the Rywal plants, the contents of free SA and its conjugates, SAGs, were measured in upper noninoculated leaves and in inoculated leaves following the mock and virus treatments. The level of salicylates in the mock-inoculated leaves showed no significant changes throughout the study, with fluctuations between 300 and 400ng (g fresh weight)^–1^ (Fig. 2B, left). In contrast, in the PVY-inoculated leaves, the salicylate levels rose between 1 and 2 dpi, reaching approximately 600ng (g fresh weight)^–1^; they then remained in that range until at least 15 dpi (Fig. 2B, right; Supplementary Table S2). Both SA and SAGs increased *c.*2-fold, which indicated *de novo* SA synthesis. The amounts of free SA remained constantly at about half those of SAGs. In contrast, there were no significant changes in SA or SAG levels in upper noninoculated leaves (Fig. 2A). To confirm that SA is synthesized *de novo* during PVY infection, temperature-shift conditions were applies. Similarly to that described previously (Szajko *et al.*, 2008), in PVY-infected Rywal plants maintained at the higher temperature (28 °C), the total SA levels increased *c*.2-fold between 1 and 3 dpi (Fig. 2C). However, by transferring these plants from 28 to 20 °C at 6 dpi, there was a significant 5-fold increase in the total SA level 24h after the temperature shift (Fig. 2C, Supplementary Table S2), accompanied by rapid lesion development (data not shown). Interestingly, at 12h post temperature shift, there was a 27-fold increase in the free SA form, followed by conversion to SAGs at 24h post temperature shift (Fig. 2C). No changes in SA levels were detected in mock-treated plants after the temperature shift. This clearly indicates that, after viral infection, SA is synthesized *de novo* and further converted into the SAG storage form.

### Role of SA in viral spread and disease development in PVY-infected Rywal plants

To further investigate the role of SA in the Rywal response to PVY infection, plants expressing the *NahG* transgene that encodes a salicylate hydroxylase were generated. Expression of the *NahG* transgene was checked in 10 selected transgenic lines using Northern analysis (Supplementary Fig. S2) and four were selected for further analyses. In contrast to potato NahG plants of cv. Pentland Ivory (Sanchéz *et al.*, 2010), and similar to NahG plants of cv. Désirée (Halim *et al.*, 2007), NahG-Rywal plants showed no yellowing or spontaneous lesion formation.

To determine the effects of *NahG* expression on the accumulation of SA, a control line (transformed with an empty vector) and plants of four selected lines with high levels of *NahG* mRNA were inoculated with PVY and the SA levels were measured 6 dpi. In the control line, the SA and SAG levels and fold of induction were similar to the nontransformed cv. Rywal (Fig. 2), whereas NahG plants did not accumulate SA or SAGs to detectable levels before or after PVY inoculation (Supplementary Fig. S3), as previously reported for potato NahG plants in response to pathogens (Halim *et al.*, 2007; Sanchéz *et al.*, 2010).

**Fig. 3.**
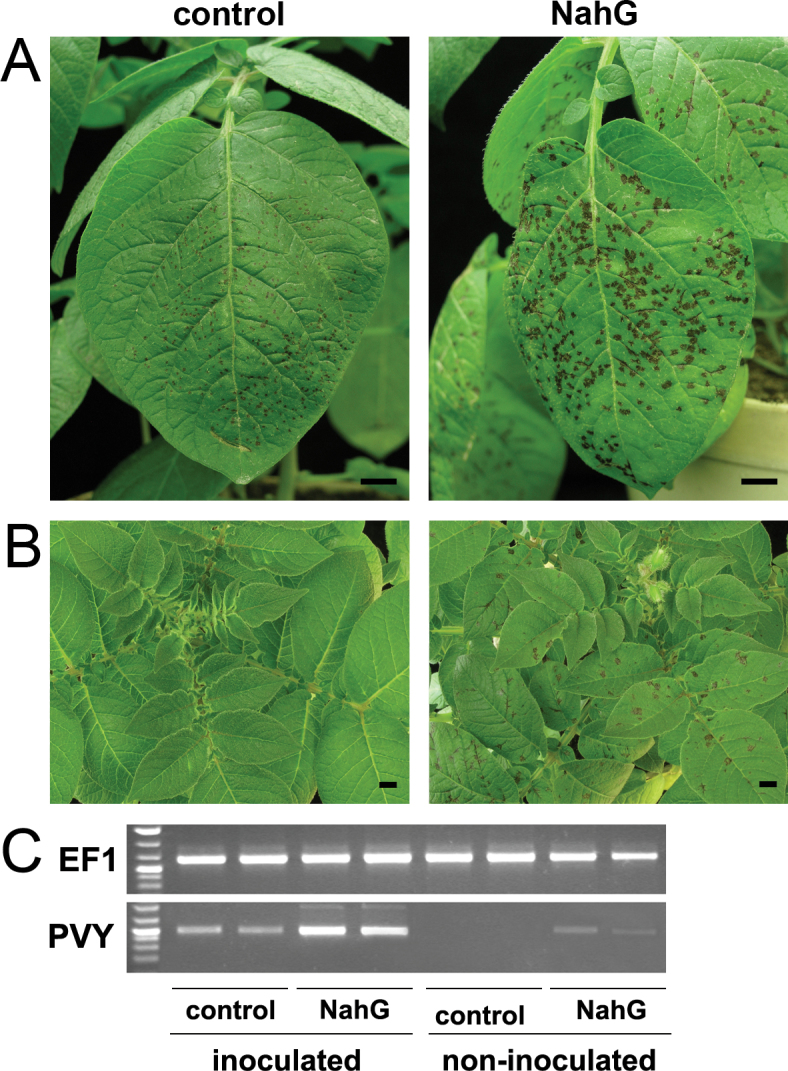
Phenotypes and viral RNA accumulation in PVY- inoculated Rywal and NahG-Rywal plants. (A, B) Symptom development on inoculated leaves in Rywal (pROK42, control) and NahG-Rywal plants at 10 d post infection (dpi) (A) and on upper noninoculated leaves at 14 dpi (B). (C) Semiquantitative reverse-transcription PCR detection of PVY coat protein RNA and *EF1* expression (loading control) in inoculated (6 dpi) and noninoculated (14 dpi) leaves, respectively. Bars: 1cm.

PVY infection of the control and NahG plants resulted in different phenotypes. In both genotypes, lesions on the inoculated leaves started to be visible by 3 dpi and developed similarly to 6 dpi. Afterwards, the lesions on the control plants remained unchanged, while those on the NahG plants continuously expanded, becoming approximately 2-fold larger at 10 dpi, in comparison to the control plants (Fig. 3A). At this time, the first signs of lesions developing on upper noninoculated leaves were observed in the NahG plants, with lesions reaching their full size at 14 dpi (Fig. 3B). At 10 dpi, the viral RNA levels were 3–4-fold higher in the inoculated leaves of the NahG plants, and at 14 dpi, viral RNA was detected in the upper noninoculated leaves of the NahG plants but not of the control plants (Fig. 3C).

To determine whether the observed changes in the sizes of the lesions and the systemic virus spreading were due to the presence or absence of SA, leaves of PVY-inoculated NahG plants were sprayed with benzothiadiazole, a functional analogue of SA. This treatment restored the normal size of the lesions, as observed for the wild-type plants (Supplementary Fig. S4A), which confirmed that the phenotype observed in the NahG plants was due to lack of SA. Moreover, to confirm that the observed enhanced susceptibility of the NahG plants was not derived from the accumulation of the SA degradation product catechol, as proposed previously (van Wees and Glazebrook, 2003), nontransformed plants were sprayed with 1mM catechol solution prior to PVY inoculation. This treatment neither changed the size of lesions nor caused lesion formation in noninoculated leaves (Supplementary Fig. S4A), which indicates that catechol itself does not induce susceptibility to PVY. The observed phenotype effects were consistent with the presence of viral RNA, which after both chemical treatments was detected only in the inoculated leaves (Supplementary Fig. S4B).

**Fig. 4.**
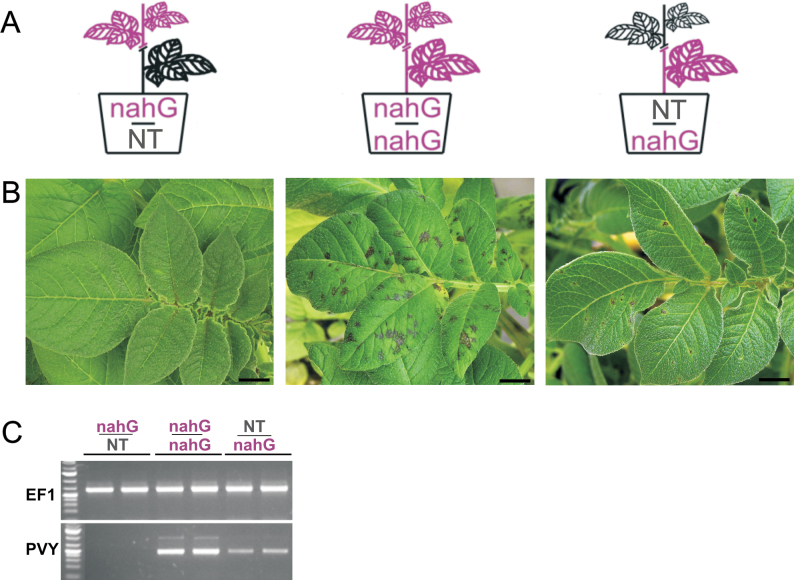
Grafting of Rywal and NahG-Rywal plants. The rootstocks of the grafted plants were inoculated with PVY. (A) Schematic representation of the grafts. (B) Scions 21 d post rootstock infection (C) Semiquantitative reverse-transcription PCR detection of PVY coat protein RNA and *EF1* expression (loading control) in all grafts. For each graft type, plant material from two independent scions was analysed. NT, nontransgenic Rywal plants; nahG, NahG-Rywal plants. Bar: 1cm.

To further investigate the role of SA in inhibition of systemic viral spread, a grafting experiment was performed with three types of grafts (Fig. 4A). When a nontransgenic scion was grafted onto a NahG stock (NT:NahG graft), systemic symptoms in the scion were observed after stock inoculation with the virus. Similarly, when a NahG plant served both as scion and rootstock (NahG:NahG graft), systemic symptoms were observed after inoculation; however, no systemic symptoms were observed when nontransgenic Rywal plants were used as rootstock (NahG:NT graft; Fig. 4B). The presence of the viral RNA in scions of NahG:NahG and NT:NahG grafts was confirmed by semiquantitative reverse-transcription PCR, whereas no virus was detected in scions of NahG:NT grafting (Fig. 4C).

### Transcriptome profiling of HR response to PVY infection

To better understand the role of SA in the *Ny-1*-mediated HR response described above, high-throughput transcriptome analysis was performed, using leaf material collected from mock- and virus-inoculated plants of cv. Rywal and NahG-Rywal at three time points (1, 3, and 6 dpi).

**Fig. 5.**
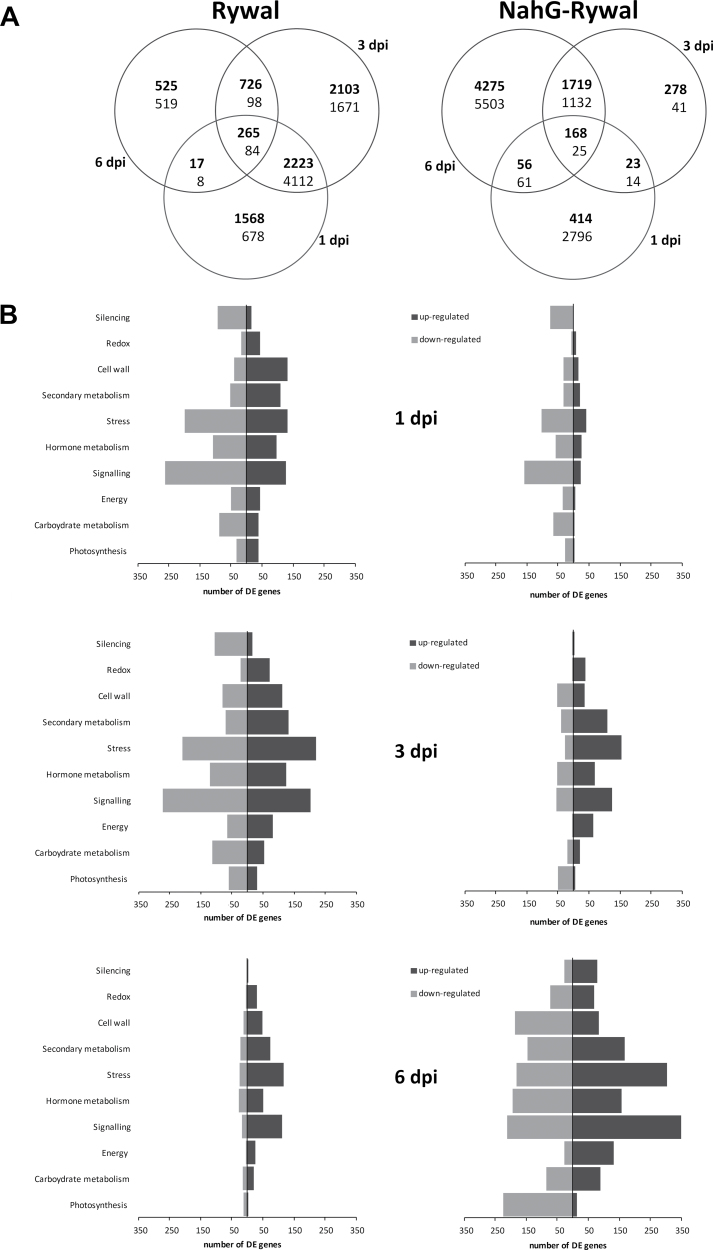
Differentially expressed (DE) genes in virus vs. mock treated plants of cv. Rywal (left) and NahG-Rywal (right) at 1, 3, and 6 d post infection (dpi). (A) Venn diagram of all of the DE genes (bold, upregulated; regular, downregulated). (B) Representation of numbers of DE genes (black, upregulated; grey, downregulated) in the selected functional groups; MapMan ontology bins: photosynthesis (bins 1, 19), carbohydrate metabolism (minor CHO, major CHO, gluconeogenesis; 2, 3, 6), energy (glycolysis, fermentation, OPP, TCA/organic transformation, mitochondrial electron transport/ATP synthesis; 4, 5, 7, 8, 9), signalling (30), hormone metabolism (17), stress (20), secondary metabolism (16), cell wall (10), redox (21), and silencing (27.5).

The plants responded to the viral inoculation by transcriptional reprogramming. In the Rywal plants, the most pronounced changes were observed at 1 and 3 dpi (Fig. 5A, left). More than 6000 genes were differentially expressed at both time points, indicating the continuity of response. Among the functional classes of the upregulated genes in Rywal at 1 dpi, there were cell-wall-, stress-, and secondary-metabolism-associated gene groups. At the same time, groups of genes associated with carbohydrate metabolism, signalling, and RNA silencing were mostly downregulated (Fig. 5B). A strong decrease in the total number of differentially expressed genes was observed at 6 dpi, where induction prevailed over repression (Fig. 5).

In contrast, in NahG plants at 1 dpi, the response was very weak, and the differentially expressed genes were mostly downregulated (e.g. stress, carbohydrate metabolism, signalling; Fig. 5). At 3 dpi, there were more upregulated genes (e.g. secondary metabolism, stress, signalling), with the exception of photosynthesis-related genes, the expression of which was strongly downregulated (Fig. 5B). Similar functional groups were differentially expressed at 6 dpi. Differential regulation of more than a quarter of all genes (Fig. 5A) could be related to disease development and multiplication of the virus at 6 dpi (Fig. 3B, C).

The microarray data were validated (correlation coefficient=0.78) by analysing 10 biologically relevant genes, involved in photosynthesis, carbohydrate, hormone metabolism, and defence response by quantitative PCR (Supplementary Table S3).

### Early activation of genes involved in defence responses

A number of genes involved in plant stress and defence responses were differentially expressed during the entire experiment in both genotypes. However, significant differences in kinetics and quantity were detected between the analysed genotypes. Induction of genes that code for pathogenesis-related proteins (PR proteins; proteinase inhibitors, thaumatin, chitinases) and genes involved in the production of ROS (mostly peroxidases), was observed at 1 dpi in both genotypes (Fig. 5B, Supplementary Table S4). However, fewer genes were differentially expressed and the differences compared to mock-inoculated plants were smaller in the SA-deficient NahG plants. On the other hand, genes coding for enzymes involved in cell-wall rearrangements and synthesis of secondary metabolites (terpenoids, flavonoids, alkaloids, simple phenolic substances) were upregulated in Rywal at 1 dpi, while in the NahG-Rywal these genes were downregulated or upregulated at later time points (Fig. 5B, Supplementary Table S4). This suggests a strong delay in gene activation in the case of SA deficiency.

Surprisingly, genes involved in signalling (several receptors and MAP kinases) were downregulated at 1 and 3 dpi in both genotypes. Moreover, the silencing components were strongly downregulated in cv. Rywal at 1 and 3 dpi (Fig. 5B, Supplementary Table S4).

### Changes in hormone signalling pathways

The shift in the overall hormonal signalling network in response to the virus in two genotypes can be best demonstrated by GSEA data (Table 1, Supplementary Table S4). In Rywal plants, the genes coding for enzymes involved in SA synthesis were upregulated at the first two time points, which correlated with increased SA (Fig. 2B). At 6 dpi, strong upregulation of genes of the jasmonic acid (JA) synthesis pathway was observed (Table 1), accompanied by upregulation of some of the genes involved in ethylene (ET) synthesis and signal transduction pathways. In the NahG plants, after the transient induction of SA synthesis pathway genes at 1 dpi, there was strong activation of the genes of both JA and ET pathways at 3 and 6 dpi. In both genotypes, there was strong inhibition of genes of the auxin signal transduction pathway (auxin responsive factors), while no changes in auxin synthesis genes were detected. Genes involved in ABA synthesis and degradation were downregulated in the NahG plants at 3 and 6 dpi. Moreover, the genes of the gibberellin and brassinosteroid pathways were induced at 1 and 3 dpi in Rywal plants, while the genes of the latter pathway were also downregulated in the NahG plants at 6 dpi.

**Table 1.** Changes in hormonal signalling Upregulated (+) and downregulated (–) MapMan functional groups identified by gene set enrichment analysis (false discovery rate corrected *Q* < 0.05) in potato cv. Rywal and NahG-Rywal at 1, 3, and 6 dpi after PVY inoculation. dpi, Days post infection.

Functional group	Rywal			NahG-Rywal		
	1 dpi	3 dpi	6 dpi	1 dpi	3 dpi	6 dpi
Salicylic acid metabolism	+	+		+		
Jasmonate metabolism			+		+	–
Ethylene metabolism					+	+
Auxin signal transduction	–	–		–	–	
ABA synthesis/degradation					–	–
Gibberellin metabolism	+	+				
Brassinosteroid synthesis/degradation	+	+				–

### Repression of photosynthesis and activation of energy-producing pathways in the disease response of the NahG plants

In Rywal plants at 1 dpi, genes involved in photosynthesis were upregulated or downregulated. Among the upregulated group, there were genes involved in light reactions (PS I and II polypeptide units) and the Calvin cycle (RuBisCO small subunit). At 3 dpi, more photosynthesis-related genes were downregulated, while at 6 dpi, only a small number of genes were differentially expressed, again mostly downregulated. The highest number of changes in gene expression were observed at 6 dpi in the NahG plants (Fig. 5A), while downregulation of photosynthesis-related genes was observed as early as 1 dpi, and these effects became significant at 3 dpi (Supplementary Table S4), and even more pronounced at 6 dpi, when many genes were downregulated (Fig. 5B). The downregulation of photosynthesis genes coincided with downregulation of tetrapyrole biosynthesis genes.

At 6 dpi in the NahG plants, there were most changes in gene expression (Fig. 5A), while the genes related to photosynthesis, the primary source of carbohydrates, were downregulated (Fig. 5B). There was strong induction of most of the genes coding for enzymes involved in glycolysis, as well as enzymes catalysing the release of glucose and fructose from sucrose (e.g. vacuolar, cytosolic, and apoplastic invertases). Phosphorylated glucose from glycolysis can enter the oxidative pentose phosphate pathway, the genes for which were also upregulated both at 3 and 6 dpi (Supplementary Table S4). The gene encoding pyruvate dehydrogenase, a regulatory point for pyruvate flux, was highly upregulated. Genes of another pathway that contributes to substrate production, lipid β-oxidation, were significantly upregulated at 3 and 6 dpi in the NahG plants (Supplementary Table S4). Strong demand for energy can also mobilize amino acids into energy pathways; not surprisingly then, there was strong upregulation of the gene encoding glutamate dehydrogenase, a connector between amino acids and the citric acid cycle (Bolton, 2009).

### Analysis of HR response to PVY at the proteome level

To complement the knowledge of the HR response of potato to PVY inoculation at the proteome level, this work analysed samples of cv. Rywal prior to the appearance of HR lesions (1 dpi, 3 dpi). Using a 2D differential gel electrophoresis approach, *c*.1200 protein spots were detected. Fifteen spots were differentially expressed after infection with PVY, in the comparison of PVY- versus mock-inoculated samples either at both time points or for individual time points. In three spots (1, 5, 9), the MS analyses indicated the presence of multiple proteins (at least one peptide matched a second nonrelated protein), and so these were excluded from further analysis. The differentially expressed proteins identified (Table 2) are involved in different metabolic pathways (e.g. in photosynthesis and different stress-response-related processes). These groups of proteins have been commonly detected as differentially expressed in previously studied plant–virus interactions (reviewed by Di Carli *et al.*, 2012). Additionally, three differentially expressed spots were detected for which the MS analysis produced only one significant peptide match (spots 8, 12) or no significant match (spot 6), and could thus not be unambiguously identified.

**Table 2.** Differentially expressed proteins in the hypersensitive resistance response of potato to infection with PVY Proteins are grouped according to their functional ontology and, for each spot, the hits identified through the NCBI databases (nr taxonomy: Viridiplantae) are given. Values are average ratio (log_2_) of the protein abundance (PVY vs. mock all together and separately for 1 and 3 dpi). dpi, Days post infection; NS, no significant change compared to control.

Spot no.	NCBI ID	Description	PVY/mock	1 dpi	3 dpi
Photosynthesis					
2	gi|108773138	RuBisCO large subunit	NS	NS	–0.8
3	gi|108773138	RuBisCO large subunit	NS	NS	–0.7
4	gi|108773138	RuBisCO large subunit	NS	NS	–0.8
13	gi|417593	RuBisCO small chain 2A	NS	NS	–0.5
15	gi|197132134	photosystem I subunit VII	NS	NS	–0.6
Plant defence (PR proteins)					
7	gi|1705808	Endochitinase 4	–2.2	NS	NS
Oxidation–reduction process (oxidative stress response)					
10	gi|228249640	Fe superoxidase dismutase	–0.7	–0.5	–0.8
14	gi|38073257	Superoxidase dismutase	NS	NS	0.4
Protein folding					
11	gi|7331143	Chaperonin 21 precursor	0.6	NS	NS
Unknown function					
6		Unidentified protein	–0.7	NS	NS
8		Unidentified protein	–0.7	NS	NS
12		Unidentified protein	NS	–0.6	NS

This work next compared the protein abundance data to the transcriptome data (Supplementary Table S5). Two superoxide dismutases were identified as differentially abundant at the protein level: one increased, while the other decreased. This result corresponds to the changes detected at the transcriptome level, albeit the response is stronger at the gene expression level (Supplementary Table S5). Similarly, protein abundance of RuBisCO small chain 2A, endochitinase 4 and chaperonin 21 matched the results at the transcript level, but the response was less intensive at the protein level and in some cases more rapid at the transcriptome level. Discrepancies in these comparisons of proteome and transcriptome data might be the consequence of time delays between the transcript and protein synthesis, or of posttranscriptional modifications that give rise to different spots on 2D gels (Yang *et al.*, 2011; Vogel and Marcotte, 2012). RuBisCO large subunit (gi|108773138) was identified in three different spots, and at the protein and transcript levels the results were diametrically opposed (i.e. upregulation at the transcript level, and downregulation at the protein level), which might be a consequence of posttranslational modifications of the protein.

## Discussion

To study the role of SA signalling in *Ny-1*-mediated HR responses to PVY, a comprehensive analysis of cv. Rywal and SA-depleted NahG-Rywal plants at the cytological, biochemical, transcriptome, and proteome levels was performed.

### Programmed cell death in the hypersensitive response to PVY

The HR is characterized by rapid and precisely localized cell death at the site of pathogen entry (Mur *et al.*, 2008). In PVY-inoculated cv. Rywal, lesions efficiently restricted the viral movement. At the cytological level, features of programmed cell death were observed. Some of these might be connected with autophagy, which is hypothesized to form a negative feedback loop in the modulation of SA signalling, to negatively regulate senescence and programmed cell death (Yoshimoto *et al.*, 2009). Other changes in ultrastructure, like chromatin condensation, have been observed previously in plant–pathogen interactions (Krzymowska *et al.*, 2007). However, no qualitative or quantitative changes in mitochondria or chloroplasts were observed in the present study (Fig. 1C), which is in contrast to previous studies (Hinrich-Berger, 1999; Pompe-Novak *et al.*, 2001; Krzymowska *et al.*, 2007).

ROS are one of the major groups of programmed cell death effectors (Mur *et al.*, 2008). In plant pathosystems, they are also involved in strengthening of the host cell walls and in cellular signalling and have thus been shown to be key elements in successful pathogen arrest (reviewed by O’Brien *et al.*, 2012). Detection of large amounts of H_2_O_2_ from 3 dpi (Fig. 1E) and the strong upregulation of peroxidase and other redox-related genes from 1 dpi, (Fig. 5B, Supplementary Tables S3 and S4) suggest that a burst of ROS has an important role in the defence response in this pathosystem.

Callose deposition has also been shown to have an important role in plant–virus interactions (Iglesias and Meins, 2000; Dobnik *et al.*, 2013; Zavaliev *et al.*, 2013). In the present study, callose deposition appeared to demarcate the lesion cells from the surrounding tissue from 3 dpi (Fig. 1F) which can be linked to the upregulation of several cell-wall-related genes as early as 1 dpi (Fig. 5B, Supplementary Table S4).

### SA is synthesized *de novo* in the *Ny-1*-dependent HR

In agreement with previous studies (Yu *et al.*, 1997), high basal levels of SA, mostly in the form of SAGs were observed in this study. In contrast to the hypothesis of Yu *et al.* (1997), that it is an enhancement of SA perception in response to pathogen attack that triggers the defence response in potato, rather than *de novo* SA synthesis, the increases in both SA forms after viral infection (Fig. 2B) suggest *de novo* synthesis of SA. This was further confirmed in temperature-shift experiments, where the increased number of virus-infected cells triggered massive *de novo* synthesis of the free form of SA (Fig. 2C). Increase of SA (Fig. 2C) at elevated temperatures, when the virus spreads systemically, suggests that SA alone is not sufficient for an effective defence, but requires additional R-gene-mediated signalling. SNC1 and N-mediated resistance has been shown to be compromised at increased temperature (Zhu *et al.*, 2010). Increased levels of salicylates have been detected in PVY-infected susceptible potato genotypes (Krečič-Stres *et al.*, 2005) and in susceptible *Arabidopsis* ecotypes upon infection with different viruses (Chandra-Shekara *et al.*, 2006; Palukaitis and Carr, 2008), which, together with the present data, could indicate that SA synthesis is, to some extent, independent of R-mediated resistance responses.

### SA deficiency causes viral spread and disease development

In agreement with previous studies on compatible (Baebler *et al.*, 2011) and incompatible (Sanchéz *et al.*, 2010) interactions between potato and viruses, the inability of Rywal plants to accumulate SA resulted in a more susceptible phenotype (Fig. 3). The most pronounced phenotypic feature of the NahG-Rywal response to viral infection was the enlargement of the lesions until at least 14 dpi. The inability of the plant to restrict the virus led to the systemic spread of the virus (Fig. 3C), which was detected also in the next generation of plants derived from the tubers of the infected plants (data not shown). Larger lesions have been reported for NahG potato plants infected with *Potato virus X* (Sanchéz *et al.*, 2010) and NahG tobacco plants infected with *Tobacco mosaic virus* (Mur *et al.*, 1997), although these studies did not shown systemic spreading of these viruses. However, SA depletion has been shown to allow *Plum pox virus* spread in tobacco (Alamillo *et al.*, 2006). In contrast, Wang and coworkers (2011) showed reductions in symptoms and viral multiplication in NahG *Arabidopsis* plants, which were possibly due to inhibition of ROS responses by higher levels of ascorbic acid and glutathione.

The data suggest that inhibition of systemic viral movement occurs at the level of cell-to-cell movement and phloem loading, as once a virus is present in the phloem, it can spread systemically even in the presence of elevated levels of SA in the plant (Fig. 4). Together with plasmodesmata-located proteins, SA has been implicated in cell-to-cell communication upon bacterial infection (Wang *et al.*, 2013). Thus, it can be concluded that the formation of lesions is a SA-independent process, although SA is a crucial element for inhibition of virus multiplication and spreading, both locally and systemically.

### Lack of SA results in quantitative differences in the transcriptome response to PVY

The whole-transcriptome analysis performed in the present study confirms the role of SA in orchestrating defence reactions. Recognition of an invading pathogen results in reprogramming of gene expression, to promote specific defence reactions in the infected plant.

The abundance and continuity of the Rywal transcriptome response at the first two time points suggests that an effective response exists already at 1 dpi, which it maintained to 3 dpi and diminished by 6 dpi (Fig. 5A). Host responses in incompatible virus interactions are more robust than those in compatible ones, most likely due to the delay in pathogen recognition or the temporal suppression of plant defence mechanisms in the compatible interactions (Jones and Dangl, 2006; Pacheco *et al.*, 2012). In NahG plants, where the same signal was sent from *Ny-1*, a weak expression response was observed at 1 dpi (Fig. 5). The lack of both the basic SA level and its increase, which can potentiate defence responses, is the most probable reason for the observed quantitative differences in the NahG response to PVY and its subsequent failure to stop viral replication and spread. Plants have different mechanisms that regulate hormone balance (Robert-Seilaniantz *et al.*, 2011). Therefore, if SA does not accumulate, then the SA-dependent mechanisms are not potentiated and the triggered response is ineffective, which leads to a shift to a different type of response in the NahG plants (Fig. 5). Differences in the extent of the gene expression changes between the genotypes (Fig. 5) can also be explained by spatiotemporal separation of the responses. As the greater part of the defence response is triggered in virus-infected tissue (Yang *et al.*, 2007), it can be speculated that the SA-driven response is localized to the lesion area, and is mostly diminished when the lesions are fully developed and the viral spread has stopped.

Plants resist pathogen infection via activation of physical and biochemical defences, such as cell-wall thickening, lignin deposition, generation of ROS, and accumulation of secondary metabolites or antimicrobial proteins, such as the PR proteins (van Loon *et al.*, 2006). Many of these processes have been shown to be SA dependent (Whitham *et al.*, 2006; Wang *et al.*, 2011). Observed differences in the defence-related responses between the genotypes used in the present study were quantitatively lower induction of PR proteins and delayed activation of cell-wall- and secondary-metabolism-related genes in the NahG-Rywal genotype, which confirms the role of SA in potentiating R-mediated responses (Shirasu and Lambas, 1997). Downregulation of cell-wall-modifying enzymes has been reported as a common feature of different plant–virus interactions (Yang *et al.*, 2007; Pacheco *et al.*, 2012). In the present study, the phenomenon has been only observed in the diseased stage (NahG.Rywal, 6 dpi). It appears that the rapid activation of cell-wall-modifying enzymes has a crucial role in preventing viral spread. The delay of the induction of the peroxidase genes (*PR-9*) might be connected with the disabled response in the NahG plants, as was shown for NahG tobacco plants (Mur *et al.*, 2000). The basal level of *PR-1* expression was two orders of magnitude lower in the NahG plants (data not shown), which indicates that SA is necessary for the maintenance of basal levels of the PR proteins, which was shown previously for NahG-Désirée potato plants (Halim *et al.*, 2007; Baebler *et al.*, 2011).

### Perturbation of hormonal signalling in the absence of SA

Complex hormonal signalling networks and their crosstalk enable efficient defence responses. It is expected that, by compromising one component, the whole hormonal regulation network might be disturbed, which was shown in this study. While the observed changes (Table 1) mostly follow the classical binary model of JA/ET- and SA-signalling antagonism (Takahashi *et al.*, 2004), the simultaneous upregulation of individual SA- and ET-signalling genes in Rywal at 3 dpi indicates possible synergistic interactions (Halim *et al.*, 2009). This also suggests that both SA- and JA/ET-dependent responses are activated upon PVY infection, but the latter are activated later and are inhibited in Rywal plants.

Auxins are known to be involved in hormone crosstalk during pathogenesis. Downregulation of several genes involved in auxin signal transduction in the present study indicates antagonistic interactions between SA and the auxins in this pathosystem. In *Arabidopsis*, auxin-responsive factors have been shown to be the major targets of viral silencing suppressors, leading to developmental abnormalities (Jay *et al.*, 2011). In contrary to the suggestion of positive correlation of JA and auxin signalling (Robert-Seilaniantz *et al.*, 2011), genes coding for auxin-responsive factors were inhibited in the NahG plants, possibly due to the synergistic action of SA and JA discussed above. Moreover, SA has been shown to negatively regulate auxin transport through inhibition of clathrin-mediated endocytosis (Du *et al.*, 2013), which might explain the downregulation of some of the auxin transport proteins (e.g. PIN7).

### Activation of energy-producing pathways in the disease response of the NahG-Rywal plants

The allocation of resources at the onset of defence reactions results in a demand for energy in the infected tissue, as the production of defence-related compounds becomes ‘top priority’ for the plant, and the photosynthetic rates are reduced until the pathogenic growth has been terminated (Bolton, 2009). This appears to be the case in the NahG plants, where the photosynthesis- and chlorophyll-biosynthesis-related genes were strongly downregulated (Fig. 5B), which is in agreement with the transcriptome data from various biotic stress studies (reviewed by Bilgin *et al.*, 2010). Interestingly, upregulation of genes involved in photosynthesis was observed at 1 and 3 dpi in Rywal plants. Local (Chou *et al.*, 2000; Berger *et al.*, 2004) and transient (Baebler *et al.*, 2009) increases in photosynthetic rates and gene expression, respectively, were observed earlier. It appears that a transient activation of photosynthesis is triggered to provide energy for initiation of the resistance response.

The collapse of photosynthesis activity inevitably leads to metabolic transition from source to sink in infected tissue. The resulting demand for carbohydrates and energy is compensated for through increased activities of hexose transporters, cell-wall invertases, the oxidative pentose phosphate pathway, and respiratory metabolism, the genes for which were all upregulated in the NahG plants at 3 and 6 dpi. This reprogramming might further enhance the expression of defence-related genes and the production of secondary metabolites (Bolton, 2009). The oxidative pentose phosphate pathway is a major source of NADPH in nonphotosynthetic cells, and NADPH oxidases are the main contributors to ROS synthesis during HR (Mur *et al.*, 2008). Interestingly, no dramatic changes in expression of genes in any of the energy-connected pathways were detected in the nontransgenic plants of cv. Rywal (Supplementary Table S4), which suggests that the response of this genotype is localized and precise, it does not lead to reprogramming of the entire plant metabolism, and it ceases when the virus is arrested.

### Conclusions

Data presented here show that *Ny-1*-mediated resistance responses can completely restrict PVY to the site of the lesion. Recognition of PVY by *Ny-1* generates a signal that initiates the plant defence response. This requires an increase in SA levels, to induce rapid changes in gene expression and to limit the viral spread. Virus inoculation induces *de novo* SA synthesis, which has a crucial role in the inhibition of PVY spreading into the parenchymal tissue. However, once the virus enters the plant vascular system, elevated levels of SA are not sufficient to stop it from spreading. SA deficiency of NahG plants results in attenuation of the plant defence responses and the virus can spread systemically.

The transcriptome analysis followed in the present study demonstrates many fascinating possibilities for interactions between both recognized and potential components of plant responses to viral infection. However, the exact signalling pathways and the effector components responsible for the changes observed still need to be elucidated.

## Supplementary material

Supplementary data are available at *JXB* online.

Supplementary Table S1. Quantitative PCR primers and probes used for microarray validation

Supplementary Table S2. Significance of changes in salicylates contents

Supplementary Table S3. Validation of microarray results by quantitative PCR.

Supplementary Table S4. Results of the gene set enrichment analysis (available as separate.xls file)

Supplementary Table S5. Comparison of transcriptome and proteome results.

Supplementary Fig. S1. PVY particles in lesion extracts

Supplementary Fig. S2. The expression of NahG in NahG-Rywal transgenic and control potato lines

Supplementary Fig. S3. Accumulation of salicylic acid and its glucoside in NahG lines

Supplementary Fig. S4. Symptom development and viral RNA accumulation after PVY inoculation and treatment with benzothiadiazole or catechol

Supplementary Data

## References

[CIT0001] Alamillo JM Saénz P García JA 2006 Salicylic acid-mediated and RNA-silencing defense mechanisms cooperate in the restriction of systemic spread of plum pox virus in tobacco. The Plant Journal 48, 217–227 17018032 10.1111/j.1365-313X.2006.02861.x

[CIT0002] Baebler Š Krečič-Stres H Rotter A 2009 PVY^NTN^ elicits a diverse gene expression response in different potato genotypes in the first 12h after inoculation. Molecular Plant Pathology 10, 263–275 19236574 10.1111/j.1364-3703.2008.00530.x PMC6640473

[CIT0003] Baebler Š Stare K Kovač M 2011 Dynamics of responses in compatible potato–Potato virus Y interaction are modulated by salicylic acid. PloS One 6, e29009 22194976 10.1371/journal.pone.0029009 PMC3237580

[CIT0004] Berger S Papadopoulos M Schreiber U Kaiser W Roitsch T 2004 Complex regulation of gene expression, photosynthesis and sugar levels by pathogen infection in tomato. Physiologia Plantarum 122, 419–428

[CIT0005] Bilgin DD Zavala JA Zhu J Clough SJ Ort DR DeLucia EH 2010 Biotic stress globally downregulates photosynthesis genes. Plant, Cell and Environment 33, 1597–1613 10.1111/j.1365-3040.2010.02167.x 20444224

[CIT0006] Bolton MD 2009 Primary metabolism and plant defense-fuel for the fire. Molecular Plant–Microbe Interactions 22, 487–497 19348567 10.1094/MPMI-22-5-0487

[CIT0007] Chandra-Shekara AC Gupte M Navarre D Raina S Raina R Klessig D Kachroo P 2006 Light-dependent hypersensitive response and resistance signaling against Turnip Crinkle Virus in *Arabidopsis* . The Plant Journal 45, 320–334 16412080 10.1111/j.1365-313X.2005.02618.x

[CIT0008] Chen H Nelson RS Sherwood JL 1994 Enhanced recovery of transformants of *Agrobacterium tumefaciens* after freeze-thaw transformation and drug selection. Biotechniques 16, 664–8, 670. 8024787

[CIT0009] Chou HM Bundock N Rolfe SA Scholes JD 2000 Infection of *Arabidopsis thaliana* leaves with *Albugo candida* (white blister rust) causes a reprogramming of host metabolism. Molecular Plant Pathology 1, 99–113 20572957 10.1046/j.1364-3703.2000.00013.x

[CIT0010] Chrzanowska M 1991 New isolates of the necrotic strain of Potato virus Y (PVYN) found recently in Poland. Potato Research 34, 179–182

[CIT0011] Di Carli M Benvenuto E Donini M 2012 Recent Insights into plant–virus interactions through proteomic analysis. Journal of Proteome Research 11, 4765–4780 22954327 10.1021/pr300494e

[CIT0012] Dobnik D Baebler Š Kogovšek P Pompe-Novak M Štebih D Panter G Janež N Morisset D Žel J Gruden K 2013 β-1,3-glucanase class III promotes spread of PVY^NTN^ and improves *in planta* protein production. Plant Biotechnology Reports 7, 547–555 24273610 10.1007/s11816-013-0300-5 PMC3824212

[CIT0013] Du Y Tejos R Beck M Himschoot E Li H Robatzek S Vanneste S Friml J 2013 Salicylic acid interferes with clathrin-mediated endocytic protein trafficking. Proceedings of the National Academy of Sciences, USA 110, 7946–7951 10.1073/pnas.1220205110 PMC3651428 23613581

[CIT0014] Edgar R Domrachev M Lash AE 2002 Gene Expression Omnibus: NCBI gene expression and hybridization array data repository. Nucleic Acids Research 30, 207–210 11752295 10.1093/nar/30.1.207 PMC99122

[CIT0015] Halim VA Altmann S Ellinger D Eschen-Lippold L Miersch O Scheel D Rosahl S 2009 PAMP-induced defense responses in potato require both salicylic acid and jasmonic acid. The Plant Journal 57, 230–242 18801014 10.1111/j.1365-313X.2008.03688.x

[CIT0016] Halim VA Eschen-Lippold L Altmann S Birschwilks M Scheel D Rosahl S 2007 Salicylic acid is important for basal defense of *Solanum tuberosum* Against *Phytophthora infestans* . Molecular Plant–Microbe Interactions 20, 1346–1352 17977146 10.1094/MPMI-20-11-1346

[CIT0017] Hennig J Malamy J Grynkiewicz G Indulski J Klessig DF 1993 Interconversion of the salicylic acid signal and its glucoside in tobacco. The Plant Journal 4, 593–600 8252063 10.1046/j.1365-313x.1993.04040593.x

[CIT0018] Hinrich-Berger J 1999 Cytological responses of susceptible and extremely resistant potato plants to inoculation with Potato virus Y. Physiological and Molecular Plant Pathology 55, 143–150

[CIT0019] Huang Z Yeakley JM Garcia EW Holdridge JD Fan JB Whitham SA 2005 Salicylic acid-dependent expression of host genes in compatible *Arabidopsis*–virus interactions. Plant Physiology 137, 1147–1159 15728340 10.1104/pp.104.056028 PMC1065414

[CIT0020] Iglesias VA Meins F Jr 2000 Movement of plant viruses is delayed in a beta-1,3-glucanase-deficient mutant showing a reduced plasmodesmatal size exclusion limit and enhanced callose deposition. The Plant Journal 21, 157–166 10743656 10.1046/j.1365-313x.2000.00658.x

[CIT0021] Ishihara T Sakurai N Sekine KT Hase S Ikegami M Shibata D Takahashi H 2004 Comparative analysis of expressed sequence tags in resistant and susceptible ecotypes of *Arabidopsis thaliana* infected with cucumber mosaic virus. Plant Cell Physiology 45, 470–480 15111722 10.1093/pcp/pch057

[CIT0022] Jay F Wang Y Yu A Taconnat L Pelletier S Colot V Renou JP Voinnet O 2011 Misregulation of AUXIN RESPONSE FACTOR 8 underlies the developmental abnormalities caused by three distinct viral silencing suppressors in *Arabidopsis* . PLoS Pathogens 7, e1002035 21589905 10.1371/journal.ppat.1002035 PMC3093370

[CIT0023] Jones JD Dangl JL 2006 The plant immune system. Nature 444, 323–329 17108957 10.1038/nature05286

[CIT0024] Jovel J Walker M Sanfaçon H 2011 Salicylic acid-dependent restriction of tomato ringspot virus spread in tobacco is accompanied by a hypersensitive response, local RNA silencing, and moderate systemic resistance. Molecular Plant–Microbe Interactions 24, 706–718 21281112 10.1094/MPMI-09-10-0224

[CIT0025] Kloosterman B De Koeyer D Griffiths R 2008 Genes driving potato tuber initiation and growth: identification based on transcriptional changes using the POCI array. Functional and Integrative Genomics 8, 329–340 18504629 10.1007/s10142-008-0083-x

[CIT0026] Kogovšek P Ravnikar M 2013 Physiology of the potato–Potato virus Y interaction. In: Lüttge U Beyschlag W Francis D Cushman J, editors, Progress in Botany 74. Berlin, Heidelberg: Springer Berlin Heidelberg

[CIT0027] Krečič-Stres H Vučak C Ravnikar M Kovač M 2005 Systemic Potato virus Y^NTN^ infection and levels of salicylic and gentisic acids in different potato genotypes. Plant Pathology 54, 441–447

[CIT0028] Krzymowska M Konopka-Postupolska D Sobczak M Macioszek V Ellis BE Hennig J 2007 Infection of tobacco with different *Pseudomonas syringae* pathovars leads to distinct morphotypes of programmed cell death. The Plant Journal 50, 253–264 17355437 10.1111/j.1365-313X.2007.03046.x

[CIT0029] Lewsey M Palukaitis P Carr JP 2009 Plant–virus interactions: defence and counter-defence. In: Parker J, editor, Molecular aspects of plant disease resistance. Wiley-Blackwell, Oxford pp 134–176

[CIT0030] Mac A Krzymowska M Barabasz A Hennig J 2004 Transcriptional regulation of the gluB promoter during plant response to infection. Cellular and Molecular Biology Letters 9, 843–853 15647801

[CIT0031] Malamy J Carr JP Klessig DF Raskin I 1990 Salicylic acid: a likely endogenous signal in the resistance response of tobacco to viral infection. Science 250, 1002–1004 17746925 10.1126/science.250.4983.1002

[CIT0032] Malamy J Hennig J Klessig DF 1992 Temperature-dependent induction of salicylic acid and its conjugates during the resistance response to tobacco mosaic virus infection. The Plant Cell 4, 359–366 12297650 10.1105/tpc.4.3.359 PMC160135

[CIT0033] Marathe R Guan Z Anandalakshmi R Zhao H Dinesh-Kumar SP 2004 Study of *Arabidopsis thaliana* resistome in response to cucumber mosaic virus infection using whole-genome microarray. Plant Molecular Biology 55, 501–520 15604696 10.1007/s11103-004-0439-0

[CIT0034] Mur LA Brown IR Darby RM Bestwick CS Bi YM Mansfield JW Draper J 2000 A loss of resistance to avirulent bacterial pathogens in tobacco is associated with the attenuation of a salicylic acid-potentiated oxidative burst. The Plant Journal 23, 609–621 10972887 10.1046/j.1365-313x.2000.00825.x

[CIT0035] Mur L Kenton P Lloyd AJ Ougham H Prats E 2008 The hypersensitive response; the centenary is upon us but how much do we know? Journal of Experimental Botany 59, 501–520 18079135 10.1093/jxb/erm239

[CIT0036] Mur LAJ Bi YM Darby RM Firek S Draper J 1997 Compromising early salicylic acid accumulation delays the hypersensitive response and increases viral dispersal during lesion establishment in TMV-infected tobacco. The Plant Journal 12, 1113–1126 9418052 10.1046/j.1365-313x.1997.12051113.x

[CIT0037] Navarre DA Mayo D 2004 Differential characteristics of salicylic acid-mediated signaling in potato. Physiological and Molecular Plant Pathology 64, 179–188

[CIT0038] O’Brien J Daudi A Butt VS Bolwell GP 2012 Reactive oxygen species and their role in plant defence and cell wall metabolism. Planta 236, 765–779 22767200 10.1007/s00425-012-1696-9

[CIT0039] Pacheco R García-Marcos A Manzano A de Lacoba MG Camañes G García-Agustín P Díaz-Ruíz JR Tenllado F 2012 Comparative analysis of transcriptomic and hormonal responses to compatible and incompatible plant–virus interactions that lead to cell death. Molecular Plant–Microbe Interactions 25, 709–723 22273391 10.1094/MPMI-11-11-0305

[CIT0040] Palukaitis P Carr JP 2008 Plant resistance responses to viruses. Journal of Plant Pathology 90, 153–171

[CIT0041] Peterhansel C Freialdenhoven A Kurth J Kolsch R Schulze-Lefert P 1997 Interaction analyses of genes required for resistance responses to powdery mildew in barley reveal distinct pathways leading to leaf cell death. The Plant Cell 9, 1397–1409 12237388 10.1105/tpc.9.8.1397 PMC157006

[CIT0042] Pompe-Novak M Wrischer M Ravnikar M 2001 Ultrastructure of chloroplasts in leaves of potato plants infected by Potato virus Y^NTN^ . Phyton 41, 215–226

[CIT0043] Postnikova O Nemchinov LG 2012 Comparative analysis of microarray data in *Arabidopsis* transcriptome during compatible interactions with plant viruses. Virology Journal 9, 101 22643110 10.1186/1743-422X-9-101 PMC3430556

[CIT0044] R Core Team 2012 R: a language and environment for statistical computing. Vienna, Austria: R Foundation for Statistical Computing

[CIT0045] Renaut J Hausman JF Bassett C Artlip T Cauchie HM Witters E Wisniewski M 2008 Quantitative proteomic analysis of short photoperiod and low-temperature responses in bark tissues of peach (*Prunus persica* L. Batsch). Tree Genetics and Genomes 4, 589–600

[CIT0046] Robert-Seilaniantz A Grant M Jones JD 2011 Hormone crosstalk in plant disease and defense: more than just jasmonate-salicylate antagonism. Annual Review of Phytopathology 49, 317–343 10.1146/annurev-phyto-073009-114447 21663438

[CIT0047] Rodrigo G Carrera J Ruiz-Ferrer V del Toro FJ Llave C Voinnet O Elena SF 2012 A meta-analysis reveals the commonalities and differences in *Arabidopsis thaliana* response to different viral pathogens. PloS One 7, e40526 22808182 10.1371/journal.pone.0040526 PMC3395709

[CIT0048] Rotter A Usadel B Baebler Š Stitt M Gruden K 2007 Adaptation of the MapMan ontology to biotic stress responses: application in solanaceous species. Plant Methods 3, 10 17784939 10.1186/1746-4811-3-10 PMC2018691

[CIT0049] Sanchéz G Gerhardt N Siciliano F Vojnov A Malcuit I Marano MR 2010 Salicylic acid is involved in the Nb-mediated defense responses to Potato virus X in *Solanum tuberosum* . Molecular Plant–Microbe Interactions 23, 394–405 20192827 10.1094/MPMI-23-4-0394

[CIT0050] Scholthof KB Adkins S Czosnek H 2011 Top 10 plant viruses in molecular plant pathology. Molecular Plant Pathology 12, 938–954 22017770 10.1111/j.1364-3703.2011.00752.x PMC6640423

[CIT0051] Shirasu K Lambas C 1997 Salicylic acid potentiates an agonist-dependent gain control that amplifies pathogen signals in the activation of defense mechanisms. The Plant Cell 9, 261–270 9061956 10.1105/tpc.9.2.261 PMC156916

[CIT0052] Singh RP Valkonen JPT Gray SM Boonham N Jones RAC Kerlan C Schubert J 2008 Discussion paper: The naming of Potato virus Y strains infecting potato. Archives of Virology 153, 1–13 17943395 10.1007/s00705-007-1059-1

[CIT0053] Smyth GK 2005 Limma: linear models for microarray data. In: Gentelman R Carey S Dudoit S Irizarry R Huber W, editors, Bioinformatics and computational biology solutions using R and Bioconductor , New York: Springer, pp. 397–420

[CIT0054] Subramanian A Tamayo P Mootha VK 2005 Gene set enrichment analysis: a knowledge-based approach for interpreting genome-wide expression profiles. Proceedings of the National Academy of Sciences, USA 102, 15545–15550 10.1073/pnas.0506580102 PMC1239896 16199517

[CIT0055] Szajko K Chrzanowska M Witek K Strzelczyk-Żyta D Zagórska H Gebhardt C Hennig J Marczewski W 2008 The novel gene *Ny-1* on potato chromosome IX confers hypersensitive resistance to Potato virus Y and is an alternative to Ry genes in potato breeding for PVY resistance. Theoretical and Applied Genetics 116, 297–303 17985110 10.1007/s00122-007-0667-1 PMC2755788

[CIT0056] Takahashi H Kanayama Y Zheng MS Kusano T Hase S Ikegami M Shah J 2004 Antagonistic interactions between the SA and JA signaling pathways in *Arabidopsis* modulate expression of defense genes and gene-for-gene resistance to cucumber mosaic virus. Plant Cell Physiology 45, 803–809 15215516 10.1093/pcp/pch085

[CIT0057] Talarczyk A Krzymowska M Borucki W Hennig J 2002 Effect of yeast CTA1 gene expression on response of tobacco plants to tobacco mosaic virus infection. Plant Physiology 129, 1032–1044 12114558 10.1104/pp.010960 PMC166498

[CIT0058] Thordal-Christensen H Zhang Z Wei Y Collinge DB 1997 Subcellular localization of H_2_O_2_ in plants. H_2_O_2_ accumulation in papillae and hypersensitive response during the barley–powdery mildew interaction. The Plant Journal 11, 1187–1194

[CIT0059] Usadel B Poree F Nagel A Lohse M Czedik-Eysenberg A Stitt M 2009 A guide to using MapMan to visualize and compare Omics data in plants: a case study in the crop species, Maize. Plant, Cell and Environment 32, 1211–1229 10.1111/j.1365-3040.2009.01978.x 19389052

[CIT0060] van Loon LC Rep M Pieterse CMJ 2006 Significance of inducible defense-related proteins in infected plants. Annual Review of Phytopathology 44, 135–162 10.1146/annurev.phyto.44.070505.143425 16602946

[CIT0061] van Wees SC Glazebrook J 2003 Loss of non-host resistance of *Arabidopsis* NahG to *Pseudomonas syringae* pv. *phaseolicola* is due to degradation products of salicylic acid. The Plant Journal 33, 733–742 12609045 10.1046/j.1365-313x.2003.01665.x

[CIT0062] Varet A Parker J Tornero P Nass N Nurnberger T Dangl JL Scheel D Lee J 2002 *NHL25* and *NHL3*, two NDR1/HIN1–1ike genes in *Arabidopsis thaliana* with potential role(s) in plant defense. Molecular Plant–Microbe Interactions 15, 608–616 12059109 10.1094/MPMI.2002.15.6.608

[CIT0063] Vogel C Marcotte EM 2012 Insights into the regulation of protein abundance from proteomic and transcriptomic analyses. Nature Reviews in Genetics 13, 227–232 10.1038/nrg3185 PMC3654667 22411467

[CIT0064] Vogel J Somerville S 2000 Isolation and characterization of powdery mildew-resistant *Arabidopsis* mutants. Proceedings of the National Academy of Sciences, USA 97, 1897–1902 10.1073/pnas.030531997 PMC26533 10677553

[CIT0065] Vuorinen AL Gammerlgard E Auvien P Somervuo P Dere S Valkonen JPT 2010 Factors underpinning the responsiveness and higher levels of virus resistance realised in potato genotypes carrying virus-specific R genes. Annals of Applied Biology 157, 229–241

[CIT0066] Wang SD Zhu F Yuan S 2011 The roles of ascorbic acid and glutathione in symptom alleviation to SA-deficient plants infected with RNA viruses. Planta 234, 171–181 21394469 10.1007/s00425-011-1391-2

[CIT0067] Wang X Sager R Cui W Zhang C Lu H Lee JY 2013 Salicylic acid regulates plasmodesmata closure during innate immune responses in *Arabidopsis* . The Plant Cell 25, 2315–2329 23749844 10.1105/tpc.113.110676 PMC3723628

[CIT0068] Whitham SA Yang C Goodin MM 2006 Global impact: elucidating plant responses to viral infection. Molecular Plant–Microbe Interactions 19, 1207–1215 17073303 10.1094/MPMI-19-1207

[CIT0069] Yang C Guo R Jie F Nettleton D Peng J Carr T Yeakley JM Fan JB Whitham SA 2007 Spatial analysis of *Arabidopsis thaliana* gene expression in response to turnip mosaic virus infection. Molecular Plant–Microbe Interactions 20, 358–370 17427806 10.1094/MPMI-20-4-0358

[CIT0070] Yang H Huang Y Zhi H Yu D 2011 Proteomics-based analysis of novel genes involved in response toward soybean mosaic virus infection. Molecular Biology Reports 38, 511–521 20373035 10.1007/s11033-010-0135-x

[CIT0071] Yoshimoto K Jikumaru Y Kamiya Y Kusano M Consonni C Panstruga R Ohsumi Y Shirasu K 2009 Autophagy negatively regulates cell death by controlling NPR1-dependent salicylic acid signaling during senescence and the innate immune response in *Arabidopsis* . The Plant Cell 21, 2914–2927 19773385 10.1105/tpc.109.068635 PMC2768913

[CIT0072] Yu DQ Liu YD Fan BF Klessig DF Chen ZX 1997 Is the high basal level of salicylic acid important for disease resistance in potato? Plant Physiology 115, 343–349 12223812 10.1104/pp.115.2.343 PMC158492

[CIT0073] Zavaliev R Levy A Gera A Epel BL 2013 Subcellular dynamics and role of *Arabidopsis* beta-1,3-glucanases in cell-to-cell movement of tobamoviruses. Molecular Plant–Microbe Interactions 26, 1016–30 23656331 10.1094/MPMI-03-13-0062-R

[CIT0074] Zhu Y Qian W Hua J 2010 Temperature modulates plant defense responses through NB-LRR proteins. PLoS pathogens 6, e1000844 20368979 10.1371/journal.ppat.1000844 PMC2848567

