# Supplementary material for: Salicylic acid is an indispensable component of the Ny-1 resistance-gene-mediated response against Potato virus Y infection in potato
Source: J Exp Bot. 2014 Jan 13;65(4):1095–109. doi: 10.1093/jxb/ert447 (PMC3935562; doi:10.1093/jxb/ert447)
Supplement: Supplementary Data [file supp_65_4_1095__index.html]

Salicylic acid is an indispensable component of the Ny-1 resistance-gene-mediated response against Potato virus Y infection in potato — Salicylic acid is an indispensable component of the Ny-1 resistance-gene-mediated response against Potato virus Y infection in potato — Supplementary Data 

# Salicylic acid is an indispensable component of the *Ny-1* resistance-gene-mediated response against *Potato virus Y* infection in potato

## Supplementary Data

Data files

**Files in this Data Supplement:**

- Supplementary Data - Supplementary Data
- Supplementary Data - Supplementary Data
