# Supplementary material for: Salicylic acid is an indispensable component of the Ny-1 resistance-gene-mediated response against Potato virus Y infection in potato
Source: J Exp Bot. 2014 Jan 13;65(4):1095–109. doi: 10.1093/jxb/ert447 (PMC3935562; doi:10.1093/jxb/ert447)
Supplement: Supplementary Data [file supp_ert447_jexbot105015_file001.pdf]

Journal of Experimental Botany

Salicylic acid is an indispensable component of the Ny-1 resistance-gene-mediated response against *Potato virus Y* infection in potato

Š. Baebler, K. Witek, M. Petek, K. Stare, M. Tušek Žnidarič, M Pompe Novak, J. Renaut, K. Szajko, D. Strzelczyk-Żyta, W. Marczewski, K. Morgiewicz, K., Gruden, J. Hennig

Supplementary Data

Supplementary data are available at *JXB* online.

- Supplementary Table S1: qPCR primers and probes used for microarray validation
- Supplementary Table S2: Significance of changes in salicylates contents
- Supplementary Table S3: Validation of microarray results by qPCR.
- Supplementary Table S4: Gene set enrichment (GSEA) analysis results (available as separate .xls file)
- Supplementary Table S5: Comparison of transcriptome and proteome results.

- Supplementary Figure S1: PVY particles in lesion extracts
- Supplementary Figure S2: The expression of *NahG* in NahG-Rywal transgenic and control potato lines.
- Supplementary Figure S3: Accumulation of salicylic acid (SA) and its glucoside (SAG) in NahG lines.
- Supplementary Figure S4. Symptom development and viral RNA accumulation after PVY inoculation and treatment with BTH or catechol

Supplementary Table S1: qPCR primers and probes used for microarray validation

Microarray results were validated by qPCR analysis of 10 biologically relevant genes. Gene functional group, name and abbreviation, primer and probe sequences and POCI/ PGSC gene identifiers are shown. Target sequences were selected by aligning target POCI genes (http://pgrc-35.ipk-gatersleben.de/poci) with genes in other databases. Primers and probes were designed in-house or by Applied Biosystems.

Group	Gene name	Primer/probe sequence (5' – 3')		Target gene (POCI / PGSC)
Carbohydrate metabolism	Phosphofructokinase (*PFK*)	FP:<br>RP:<br>MGB probe:	CTACAGTTCTGTAATCTCAATGTGTGAGT<br>GCATTTATCTATAGTATTATTTAGAGTCACAAAATACACTT<br>AAGCTTGGTGAGTTTTAC	MICRO.4320.C1<br>PGSC0003DMG400045386
	Citrate synthase (*CS*)	FP:<br>RP:<br>MGB probe:	GCCAATCAGGAAGTTTTGCTATGGA<br>ACATAGTCTTTCAACTGCTCTTTGGAA<br>TCTCCCCACATTCTTC	MICRO.885.C1<br>PGSC0003DMG400028982
	chlorophyll a-b binding protein 4 (*CAB4*)	Kogovšek et al., 2010		MICRO.4180.C3<br>PGSC0003DMG400006149
Hormonal signaling	Lipoxygenase (*9-LOX*)	FP:<br>RP:<br>MGB probe:	TCCCTACAAGTGAACAAGGACTCA<br>ACTTGACGATGTGGAGATTTAAATGAAAGT<br>CAGGCAAAGGAATACC	MICRO.11738.C1<br>PGSC0003DMG400010859
	Acyl-CoA oxidase (*ACX3*)	FP:<br>RP:<br>MGB probe:	GGCATGGAAGTAATGTTCGAGGTAT<br>ACTTTTGAGCTGATTCACATGGAGTATT<br>CCTGTGCTTGAATCAT	MICRO.7247.C1<br>PGSC0003DMG400010548
	Auxin response factor 2 (*ARF2*)	FP:<br>RP:<br>MGB probe:	CCAGTCATAGCATGCATCTTGGT<br>TGGTTTGTAATACACAGTGAACATCGT<br>CCAAGCTGTTGCAAGTAC	MICRO.5396.C1<br>PGSC0003DMG400014179
	UDP-glycosyltransferase, *UDP-GTF*	FP:<br>RP:<br>MGB probe:	AGAGAATGCAATGAAATGGAAAAAATTAGCT<br>TTTAGTGCATTACAAGTTTGAAAGAAATTCTTCA<br>CTCCCTCCTTCATCTACTG	MICRO.10580.C3<br>PGSC0003DMG400029349
Defence responses	Peroxidase precursor (*POX*)	FP:<br>RP:<br>MGB probe:	GGTTACGATTTTTTTTATTCTCGTGCTAGT<br>GTACTGGAATAAAATCCAACACGAGTC<br>CCTTGGCCGAATACC	MICRO.11901.C2<br>PGSC0003DMG400012589
	Pathogenesis-related protein 1b (*PR1-b*)	Baebler et al, 2011		MICRO.8918.C2<br>PGSC0003DMG400002027<br>PGSC0003DMG400002028<br>PGSC0003DMG400002029
	β-1,3-glucanase II (*Glu II*)	Kogovšek et al., 2010		MICRO.2286.C42<br>PGSC0003DMG401010492<br>PGSC0003DMG400010490

Supplementary Table S2: Significance of changes in salicylates contents

Significances of changes (t-test; ***: p<0.001, **: p<0.01, *: p< 0.05, NS: not significant) in SA and SAG levels in (A) upper, non–inoculated (relates to Fig. 2A) and inoculated (relates to Fig. 2B) in leaves of potato cv. Rywal from 0 to 15 dpi are shown for comparisons between virus and mock-inoculated samples (PVY vs. mock), and from the first time point to other time points (0dpi vs. other time points) and in (B) temperature shift experiment (relates to bar graph in Fig. 2C) in PVY inoculated leaves of cv. Rywal 12 and 24 hours after temperature shift from 28°C to 20°C preformed at 6 dpi.

**A**			**0 dpi**	**1 dpi**	**2 dpi**	**3 dpi**	**6 dpi**	**9 dpi**	**12 dpi**	**15 dpi**
non-inoculated leaves	PVY vs. mock	SA	NS	NS	NS	NS	NS	NS	NS	NS
Fig 2A		SAG	NS	NS	NS	NS	NS	NS	*	*
inoculated leaves	PVY vs mock	SA	NS	NS	NS	NS	NS	**	**	NS
Fig 2B		SAG	*	NS	*	**	**	*	**	**
	mock 0dpi vs. other time points	SA	-	NS	NS	NS	NS	NS	NS	NS
		SAG	-	NS	NS	NS	*	NS	NS	NS
	PVY 0 dpi vs. other time points	SA	-	NS	*	NS	NS	**	*	NS
		SAG	-	NS	*	**	**	**	**	*

**B**			**12h**	**24h**
PVY inoculated leaves	6 dpi vs time after shift	SA	***	*
Fig 2C - bar graph		SAG	**	***

Supplementary Table S3: Validation of microarray results by qPCR.

qPCR using SYBR Green (*CAB4*, *PR–1b*, *GluII*) or TaqMan (other genes) chemistry, Cq values determination and relative gene expression quantification were performed as described earlier (Baebler et al., 2009). Log_2 of ratios between the expression in virus and mock inoculated plants of cv. Rywal and NahG-Rywal 1, 3 and 6 days after PVY inoculation obtained by microarray (M) or qPCR (P) analysis are shown. Gene full names and their POCI/PGSC IDs are shown in Supplemental table S1. Statistically significant values ($p < 0.05$) are marked with bold. Correlation coefficient between microarray and qPCR data was 0.78. Relatively lower correlation observed for *ARF2* and *ACX3* genes can be attributed to different specificity of qPCR and microarray probes due to incomplete genome sequence information.

	**Rywal 1dpi**		**Rywal 3dpi**		**Rywal 6dpi**		**NahG-Rywal 1dpi**		**NahG-Rywal 3dpi**		**NahG-Rywal 6dpi**	
**Name**	M	P	M	P	M	P	M	P	M	P	M	P
CS	**0.65**	0.13	**1.85**	**0.71**	**0.85**	**0.67**	**0.53**	0.05	**1.8**	**0.95**	**1.91**	**1.76**
PFK	0.36	0.24	**1.77**	**1.1**	**0.97**	0.61	**0.6**	**0.36**	**2.45**	**1.99**	**2.18**	**2.16**
CAB4	0.13	-0.28	-0.25	-0.37	-0.1	-0.31	**0.51**	0.3	-0.22	**-0.71**	**-0.95**	**-1.35**
UDP-GTF	**1.25**	0.57	**1.17**	-0.12	0.2	-0.37	0.39	-0.15	**-0.79**	**-0.62**	**-1.15**	**-0.65**
ARF2	**-1.39**	0.25	**-2.11**	-0.16	**-0.61**	**-0.53**	**-0.78**	0.13	**-0.71**	**-0.92**	**-1.01**	**-1.74**
ACX3	**-2.38**	-0.03	**-1.56**	**1.11**	**1.22**	**1.19**	**-1.45**	**0.27**	**1.63**	**1.38**	**3.4**	**2.41**
**LOX-9**	-0.78	-2.04	**2.25**	2.86	**3.41**	**4.97**	0.62	0.4	**4.53**	**7.53**	**5.62**	**11.09**
**POX**	-0.05	0.58	**2.52**	3.68	**4.05**	**4.42**	-0.06	0.29	**6.49**	**5.84**	**7.4**	**8.34**
PR1b	-0.81	0.53	0.96	**2.02**	**2.86**	**4.11**	0.64	0.95	**5.07**	**9.7**	**5.34**	**11.62**
GluII	**-1.61**	0.04	**2.13**	**1.5**	**2.87**	**2.66**	0.52	0.16	**5.16**	**2.14**	**5.44**	**3.1**

Supplementary Table S5: Comparison of transcriptome and proteome results.

Proteins, differently expressed in HR Rywal 1 or 3 dpi, grouped according to their functional ontology. For each spot the hits identified through the NCBI (nr taxonomy: Viridiplantae) and POCI databases search are given. As different POCI sequences can represent the same gene, allelic variant or closely related gene family multiple hits were often retrieved per one protein in this database. 2D-DIGE results are thus linked to multiple results of microarray analysis. Average ratio (log_2) of the protein abundance (PVY vs. mock all together and separately for 1 and 3 dpi) or gene expression (PVY vs. mock in each time point) is shown. NS - no significant change compared to control.

**2D-DIGE**						**Microarrays**		
**Spot No.**	**NCBI ID**	**Description**	**PVY/mock**	**1dpi**	**3dpi**	**POCI ID**	**1dpi**	**3dpi**
**Photosynthesis**								
2	gi|108773138	RuBisCO large subunit	NS	NS	-0.8	cSTB44J7TH	1.6	1.1
3	gi|108773138	RuBisCO large subunit	NS	NS	-0.7	cSTB44J7TH	1.6	1.1
4	gi|108773138	RuBisCO large subunit	NS	NS	-0.8	cSTB44J7TH	1.6	1.1
13	gi|417593	RuBisCO small chain 2A	NS	NS	-0.5	PPCBW28TH	-0.4	NS
						MICRO.4165.C2	NS	-0.5
						MICRO.4165.C5	NS	0.6
						MICRO.4165.C6	NS	NS
						MICRO.4165.C11	NS	NS
						MICRO.4165.C12	NS	NS
						PPCCK68TH	1.0	NS
						PPCBJ92TH	1.2	1.5
						MICRO.4165.C17	NS	NS
						BPLI8M18TH	NS	NS
						cSTB2C1TH	NS	NS
15	gi|197132134	photosystem I subunit VII	NS	NS	-0.6	MICRO.16458.C1	1.3	0.7
**Plant defense (PR proteins)**								
7	gi|1705808	Endochitinase 4	-2.2	NS	NS	MICRO.556.C10	-1.1	NS
						MICRO.556.C3	1.5	3.7
						MICRO.556.C7	1.2	3.1
						MICRO.556.C2	NS	1.6
						MICRO.556.C8	NS	NS
						MICRO.556.C1	NS	NS
						POCAZ84TP	NS	1.6
						MICRO.11279.C2	NS	2.5
						MICRO.11279.C1	NS	NS
						MICRO.4230.C1	0.8	2.3
						MICRO.10439.C4	-0.9	NS
**Oxidation-reduction process (oxidative stress response)**								
10	gi|228249640	Fe superoxidase dismutase	-0.7	-0.5	-0.8	MICRO.1819.C1	-1.8	-2.8
						MICRO.1819.C2	NS	-0.8
						MICRO.1819.C6	NS	-2.5
						MICRO.2240.C1	1.1	1.2
						MICRO.2240.C2	1.8	1.4
						cSTE21I8TH	NS	NS
14	gi|38073257	Superoxidase dismutase	NS	NS	0.4	MICRO.4193.C1	1.0	1.4
						MICRO.4193.C2	0.7	0.8
**Protein folding**								
11	gi|7331143	Chaperonin 21 precursor	0.6	NS	NS	MICRO.115.C2	0.9	1.0
						MICRO.115.C1	NS	-0.7
						MICRO.3337.C1	NS	NS
						cPRO13C12TH	NS	NS
**Unknown function**								
6		Unidentified protein	-0.7	NS	NS			
8		Unidentified protein	-0.7	NS	NS			
12		Unidentified protein	NS	-0.6	NS			

Supplementary Figure S1: PVY particles in lesion extracts

The presence of viral particles in inoculated leaves of cv. Rywal was examined 6 days after inoculation by the negative staining method under transmission electron microscope (Philips CM 100; Amsterdam). Lesions (shown on image) and two neighbouring pieces (the first close to the lesion; the second close to the first; data not shown), each 1 mm wide, were excised from the leaf. The virus particles were extracted from each piece separately (0.1 M phosphate buffer, 2% polyvinylpyrrolidone). The suspensions (20 µL) were adsorbed onto formvar-coated, carbon stabilised copper grids and negatively stained with 1% (w/v) uranyl acetate. The grids were inspected under transmission electron microscopy, operating at 80 kV, with images recorded using a BioScan 792 camera (Gatan).

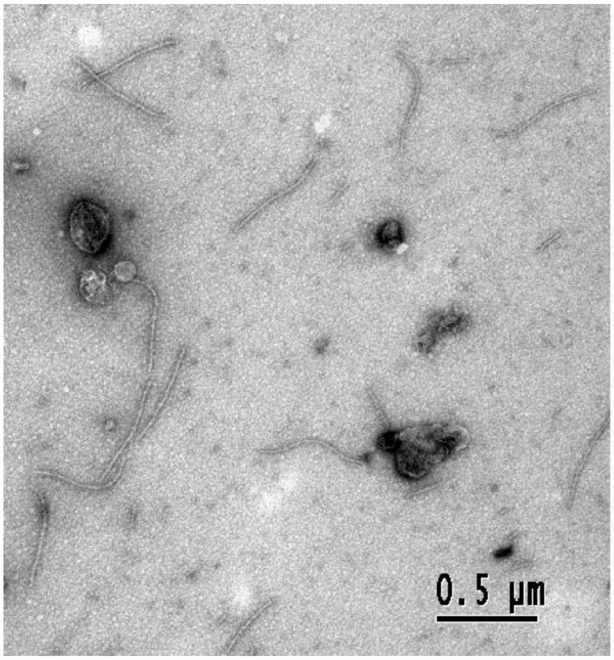

Supplementary Figure S2: The expression of *nahG* in NahG-Rywal transgenic and control potato lines.

Total plant RNA was isolated from 100 mg of frozen and homogenized plant tissue with Trizol Reagent (Invitrogen; Chomczynski and Sacchi, 1987). Primers for hybridization probe were designed with LaserGene PrimerSelect, using *Pseudomonas putida nahG* sequences (GeneBank Acc. M60055) and were UppnahG: GGCGGCATCATCAACGTGGTGGCTTTCA and LownahG: AGGGCGGCGCAGGTCGTCGTAGGCTTCA. PCR products was column-purified and labelled with [^{32}P]α–dCTP or [^{32}P]α–dATP (Hartmann) using commercially available DNA labelling kit (Fermentas) following the manufacturer's protocol. 10 µg RNA samples were separated in 1% agarose gel, blotted onto membrane and subjected to Northern hybridization with *nahG* probe (upper panel). To normalize to RNA amount, the membrane was stained with methylene blue prior to hybridization (lower panel). The numbers represent independent NahG-Rywal transgenic lines; pROK1 and pROK42 – control lines (transformed with an empty vector).

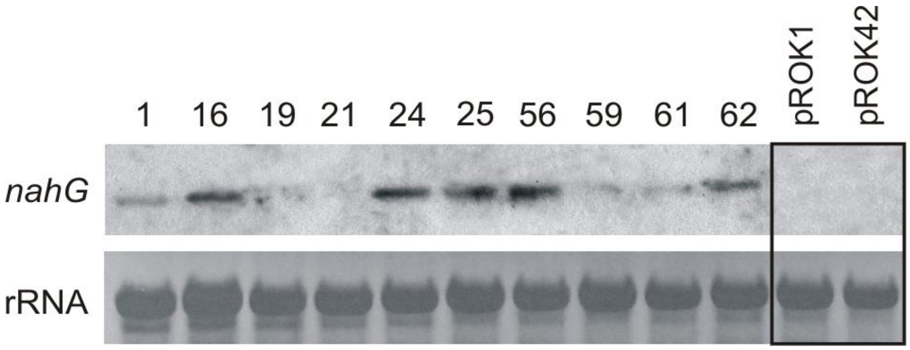

Supplementary Figure S3: Accumulation of salicylic acid (SA) and its glucoside (SAG) in NahG lines.

SA and SAG were extracted from PVY (+) or mock-inoculated (-) leaves of control (pROK42 transformed with empty vector) and four transgenic lines 6 days after PVY infection (at the time of fully developed lesions). Data are means ± standard deviation (n = 5). All changes in SA and SAG content were statistically significant (t-test p>0.01) between NahG and control lines.

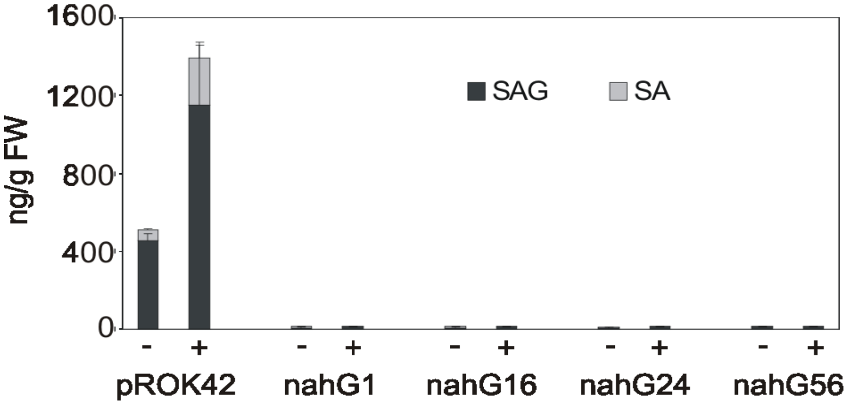

Supplementary Figure S4. Symptom development and viral RNA accumulation after PVY inoculation and treatment with BTH or catechol

Leaves of PVY-inoculated NahG-Rywal or Rywal plants were sprayed with SA functional analogue benzothiadiazole (BTH) or SA degradation product catechol (CAT), as described by Ryals et al. (1996) and Halim et al. (2007), respectively. Symptom development (A; scale bar = 1 cm) and viral RNA accumulation (B) was monitored in PVY inoculated (10 dpi; lower panels) and upper, non-inoculated (14 dpi, upper panels) leaves.

For PVY accumulation reverse transcription reactions contained 1µg DNase I - treated RNA from PVY - or mock -inoculated plants, primed with oligo(dT) and elongated by the PVY specific sequence (5'oligo(dTx30)GTCTCCTGATTGAAGTTTACAG3') and containing 200 U M-MuLV Reverse Transcriptase (Fermentas) were set to synthesize the first-strand of cDNA. PCR reactions (25 µl) to detect PVY coat protein RNA (PVYCP) contained an appropriate reaction buffer, 2.5 mM MgCl2, 0.25 mM dNTPs, specific primers (PVYCPf - CACCGTCCAACCCGAAAAG; and PVYCPr – AAATACTCGGGCAACTCAATCACA at 400 nM each). PCR was performed for 22 cycles at 94°C for 30 s, 53°C for 30 s, and 72°C for 45 s in GeneAmp 2720 Thermal Cycler (Applied Biosystems). Separate PCR reactions of elongation factor 1-α (EF-1) gene expression were performed as loading control (Varet et al., 2002).

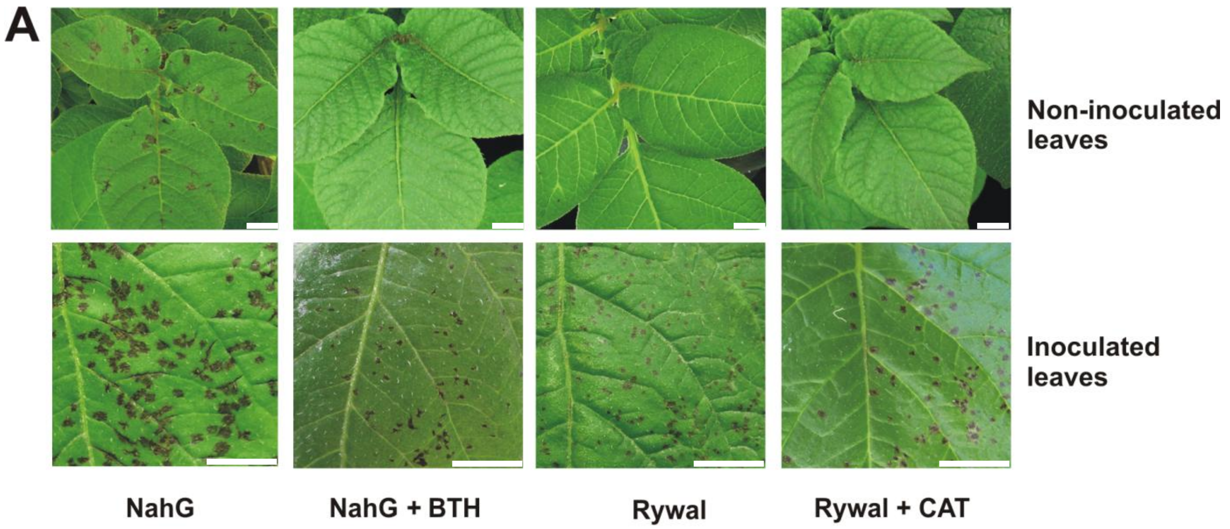

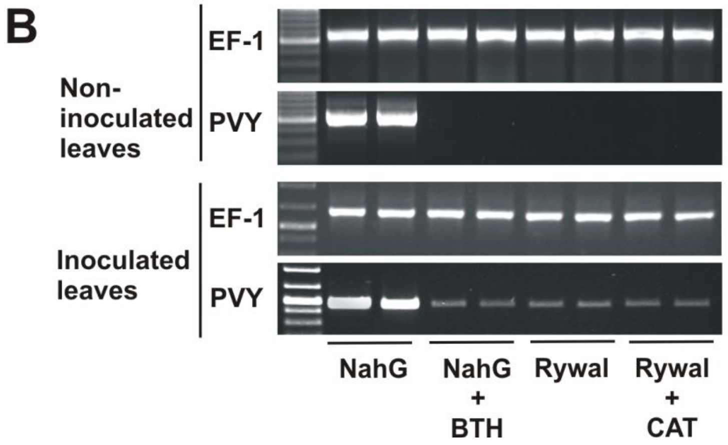

Supplementary references

Baebler Š, Krečič-Stres H, Rotter A, et al . 2009. PVY^{NTN} elicits a diverse gene expression response in different potato genotypes in the first 12 h after inoculation. Molecular Plant Pathology **10,** 263-275.

Chomczynski P, Sacchi N. 1987. Single-step method of RNA isolation by acid guanidinium thiocyanate-phenol-chloroform extraction. Analitycal Biochemistry **162,** 156-159.

Halim VA, Eschen-Lippold L, Altmann S, Birschwilks M, Scheel D, Rosahl S. 2007. Salicylic Acid Is Important for Basal Defense of Solanum tuberosum Against *Phytophthora infestans*. Molecular Plant-Microbe Interactions **20,** 1346-1352.

Kogovšek P, Pompe-Novak M, Baebler Š, Rotter A, Gow L, Gruden K, Foster GD, Boonham N, Ravnikar M. 2010. Aggressive and mild *Potato virus Y* isolates trigger different specific responses in susceptible potato plants. Plant Pathology **59**, 1121–1132.

Ryals JA, Neuenschwander UH, Willits MG, Molina A, Steiner HY, Hunt MD. 1996. Systemic Acquired Resistance. The Plant Cell **8,** 1809-1819.

Varet A, Parker J, Tornero P, Nass N, Nurnberger T, Dangl JL, Scheel D, Lee J. 2002. NHL25 and NHL3, two NDR1/HIN1-1ike genes in Arabidopsis thaliana with potential role(s) in plant defense. Mol Plant Microbe Interact **15**, 608–616.
